# Evidence Against Syntactic Encapsulation in Large Language Models

**DOI:** 10.1111/cogs.70187

**Published:** 2026-03-10

**Authors:** Thomas A. McGee, Yiyang Zhang, Idan A. Blank

**Affiliations:** ^1^ Department of Psychology University of California, Los Angeles; ^2^ Department of Linguistics University of California, Los Angeles

**Keywords:** Modularity, Syntax, Semantics, Neural networks, Large language models

## Abstract

Transformer‐based large language models (LLMs) have recently demonstrated exceptional performance in a variety of linguistic tasks. LLMs primarily combine information across words in a sentence using the attention mechanism, implemented by “attention heads:” these components assign numerical weights linking different words in the input to one another, capturing different relationships between these words. Some attention heads automatically learn to assign weights that accurately encode meaningful linguistic features including, importantly, heads that appear specialized for identifying particular syntactic dependencies. Are syntactic computations in such heads “encapsulated”, i.e., impenetrable to the influence of non‐syntactic information? Such encapsulated computations would be strikingly different from those of the human mind, where non‐syntactic information sources (e.g., semantics) influence parsing from the earliest moments of online processing, and where syntax and semantics are tightly linked in the mental lexicon. Here, we tested whether the activity of “syntax‐specialized” attention heads in transformer‐based LLMs is modulated by one type of semantic information: plausibility. In each of three LLMs (BERT, GPT‐2, and Llama 2), we first identified attention heads specialized for various dependency types; in nearly all cases tested, we then found that implausible semantic information reduces attention between the words that constitute the dependency for which a head is specialized. These results demonstrate that, even in attention heads that are the best a‐priori candidates for syntactic encapsulation, syntactic information is penetrable to semantics. These data are broadly consistent with the integration of syntax and semantics in human minds.

## 1 Introduction

Transformer‐based large language models (LLMs) have achieved remarkable performance in a variety of language tasks (e.g., Brown et al., 2020; Chang & Bergen, 2024; Contreras Kallens, Kristensen‐McLachlan, & Christiansen, 2023; OpenAI, 2022; Piantadosi, 2023; Vaswani et al., 2017). The text that LLMs generate, as well as their internal representations, indicate that they have mastered many (but not all) syntactic abstractions that underlie the structure of sentences (e.g., Diego‐Simón, D'Ascoli, Chemla, Lakretz, & King, 2024; Manning, Clark, Hewitt, Khandelwal, & Levy, 2020; McCoy, Smolensky, Linzen, Gao, & Celikyilmaz, 2023; Wilcox, Vani, & Levy, 2021; for reviews, see Linzen & Baroni, 2021; Mahowald et al., 2024; Millière, 2024). To the extent that LLMs have human‐like syntactic knowledge, they may constitute good models of (some aspects of) human language processing (Blank, 2023). However, this latter claim depends on whether LLMs, beyond “having syntactic knowledge” in the broad sense, also align with humans in the finer details of how they represent and process syntax (Arehalli, Dillon, & Linzen, 2022; Van Schijndel & Linzen, 2021).

One of the central properties of human syntactic processing is that it is rapidly influenced by external information sources, such as semantic knowledge or visual referents. Whereas some interpretations of generative linguistic theory (Chomsky, 1957, 2000, 2001), as well as some prominent views in psycholinguistics (e.g., Ferreira & Clifton, 1986; Frazier, 1987; Frazier & Fodor, 1978) and neuroscience (Friederici, 2002, 2011; Friederici & Kotz, 2003) have viewed syntax as initially impenetrable to nonsyntactic influences, the empirical evidence now suggests that syntax is not encapsulated (Bates & MacWhinney, 1987; Fromont, Steinhauer, & Royle, 2020; MacWhinney, 2014; McRae, Spivey‐Knowlton, & Tanenhaus, 1998; Steinhauer & Drury, 2012; Tanenhaus, Spivey‐Knowlton, Eberhard, & Sedivy, 1995; Trueswell, Tanenhaus, & Garnsey, 1994). Rather than proceeding independently of other processes, the mind's syntactic parser appears to opportunistically use various information sources as soon as they become available (or, at least, as soon as we can measure), which is beneficial for rapid comprehension and accurate disambiguation, reducing the need for later reprocessing. The question of encapsulation versus penetrability is fundamental in cognitive science because it is one of the main characteristics of cognitive modules^1^ (Fodor, 1983), and whether a processing domain (such as syntax) is modular is a basic architectural property of a computing system.

To demonstrate the distinction between a modular and a nonmodular syntactic parser, consider the sentence “We admired the architect of the house that was recently renovated.” From a syntactic point of view—ignoring word meaning—the sentence is structurally ambiguous, because the clause “that was recently renovated” can describe either the architect or the house (this may be clearer in the sentence “we admired the architect of the house that everyone knows,” where the relative clause “that everyone knows” is semantically compatible with the architect or the house). However, the ambiguity in the first sentence can be resolved using semantic information: architects (people) are not renovated, but houses can be. Hence, the clause “that was recently renovated” likely describes the house. A parser that is initially encapsulated would be unable to use this semantic information in its early processing stages to resolve the structural ambiguity (just like in the sentence with “that everyone knows”). For such a parser, the structure of the two sentences above would be equally ambiguous in early processing stages, prior to the integration of semantic information, which would only occur later. In contrast, a parser that is penetrable from the earliest moments would not encounter ambiguity in the first sentence, immediately determining the correct structure.

Is syntactic processing in LLMs encapsulated from nonsyntactic information sources that exist in their textual input, or can such sources penetrate syntactic computations? One may be tempted to conclude that syntax is encapsulated because these models can generate next‐word predictions that obey syntactic rules even for nonsense sentences that are “devoid of semantics” (e.g., for a stem like “the colorless green ideas that I ate with the chair…,” an LLM may still assign a higher probability to “sleep” than to “sleeps” as the next word) (Gulordava, Bojanowski, Grave, Linzen, & Baroni, 2018). Similar reasoning has been applied to the human mind (Chomsky, 1957, 1965): given that we can judge the grammaticality of such nonsense sentences, one may argue that the mental parser must be encapsulated. However, such conclusions about encapsulation do not logically follow: even if a parser were penetrable to, for example, lexico‐semantic information, we could reasonably assume that it could still process syntactic features in the absence of coherent meaning. In other words, a parser being penetrable to semantics does not entail that semantics are necessary for its proper operation.

A closer look at how LLMs process “meaningless” sentences instead suggests that syntactic processing is not encapsulated in the hidden layers, but is rather penetrable to semantics: syntactic violations are harder for LLMs to detect in meaningless sentences than in meaningful sentences (as evident in their next‐word prediction probabilities; Gulordava et al., 2018); and the quality of syntactic representations in LLM hidden layers is lower for “Jabberwocky” sentences, in which content words are replaced with nonwords with correct morphosyntactic inflection (Arps, Kallmeyer, Samih, & Sajjad, 2023; Maudslay & Cotterell, 2021). However, such findings could reflect a lack of exposure to nonwords rather than a strictly semantic influence. More generally, LLMs do not only store and process the meaning of words but might also associate some syntactic structures with their own abstract meaning,^2^ which combines with the meanings of individual words (Rozner, Weissweiler, Mahowald, & Shain, 2025; Li, Zhu, Thomas, Rudzicz, & Xu, 2022; Veenboer & Bloem, 2023). Such syntax‐semantics links are consistent with theories of linguistic representations in the human mind, namely, Construction Grammars (Goldberg, 1995, 2003, 2019), according to which syntactic objects are not merely hierarchical structures but are rather associated with their own meanings (e.g., the structure of “colorless green ideas sleep furiously” roughly means “things with a specific type of a certain property do something in a particular way”). Each attention head uses its own function to determine how the representation of a given word combines with the representations of other words. Attention weights may be computed according to various features (e.g., linear order, syntax, associated meanings, statistical co‐occurrence, coreference, etc.). The residual stream receives the outputs of all attention heads that immediately precede it and, thus, its representation of each word is likely influenced by this wide variety of relationships to other words in the sentence. Therefore, representations of syntactic structure in the residual stream are likely entangled with semantic representations.

Finding syntactic representations that appear penetrable to nonsyntactic information *somewhere* within LLMs is not surprising—during language processing, different information sources must eventually combine and interact with one another. But such penetrable computations in one part of an LLM might coexist alongside encapsulated representations in other parts. Moreover, syntactic penetrability is not a foregone conclusion: indeed, some arguments for encapsulation in the human mind claim that it is “an optimal solution” to the problem of language processing (Chomsky, 1957, 2000, 2001). If this were the case, LLMs may naturally “discover” it, similarly to artificial neural networks in the domain of vision, which have been shown to learn to dissociate different computational problems by separating them into different internal circuits (e.g., Dobs, Martinez, Kell, & Kanwisher, 2022). Namely, an initial stage of syntactic analysis that is encapsulated from external influences might prove useful for meeting an LLM's training objective of next word prediction (or missing word prediction), or may arise early during a model's training and, with further learning, build upon it rather than change it.

Therefore, claiming the syntactic computations in LLMs are not encapsulated from nonsyntactic information requires testing processing loci that are strong a priori candidates for encapsulation. If one were to look for syntactic encapsulation in LLMs, what would be a good place to start? To answer this question, consider the architecture of transformer‐based LLMs. Like other types of LLMs, transformers represent every token (usually, a word) in a sentence in a distributed manner across hidden units, and this representation is gradually transformed as it passes through the model's layers. The main feature that distinguishes transformers from alternative LM architectures such as LSTMs is that the transformation of a token's representation from one layer to the next is heavily influenced by computations in components called “attention heads” (Vaswani et al., 2017). These attention heads capture how tokens in a text input relate to one another by assigning numerical weights from each token to other tokens, directing tokens to “attend” to one another. In bidirectional transformers, each token can attend to all tokens, both preceding and following it (as well as to itself); in unidirectional transformers, each token can only attend to (itself and) all preceding tokens, but not to those following it. Each token's representation is transformed into a “mix” of all the tokens it attends to, via a weighted linear combination wherein tokens that are attended to more strongly are represented more prominently. Each attention head assigns different patterns of weights across tokens, producing a distinct “mixed” representation and capturing a different informational subspace of the input. Blocks of attention heads alternate with feedforward neural network layers, such that attention representations are combined across all heads within a block, added back to the residual stream, and then passed to the next layer for further processing.^3^

Thus, the hidden layers themselves are perhaps expected to have representations where syntactic information is somewhat entangled with nonsyntactic (e.g., semantic) information: as representations are transformed within a given layer *n*, tokens can attend to one another in diverse ways across attention heads, such that a token's resulting representation in layer *n+*1 contains information about the representations of many tokens from layer *n*, for myriad reasons, both syntactic and otherwise. In contrast, we reasoned that if encapsulated syntactic computations existed anywhere within LLMs, the best candidates for such computations would be individual attention heads: each head learns to assign unique patterns of attention weights across tokens, and some such patterns might purely target syntactic relationships between tokens (but not other types of relationships) in a manner that is impenetrable to nonsyntactic information. In other words, specialized attention heads could learn to transform embedding vectors purely based on specific syntactic content (e.g., computing high attention weights for any subject‐verb pair). Because attention heads allow for information transfer between tokens via query‐key attention, this offers a clear mechanism for how dependencies present in the input could be initialized and stored by the model. It is less clear how feedforward layers could represent syntactic structures such as dependencies that are not already present in the residual stream, because the computations in a given feedforward layer operate on each token independently without direct transfer of representational content between the embeddings of different tokens.

Even though the question of whether syntactic representations are encapsulated in feedforward layers has not been directly investigated,^4^ various studies have identified neurons specialized for syntactic information or semantic features (e.g., Durrani, Sajjad, Dalvi, & Belinkov, 2020; Suau, Zappella, & Apostoloff, 2020). However, Bolukbasi et al. (2021) found that sentences which maximally activate particular neurons are not consistent across datasets, suggesting that neurons in feedforward layers encode complex feature sets. This finding is consistent with the idea that in subsymbolic, connectionist models, distributed networks of neurons must be repurposed across a variety of inputs. This result is also consistent with recent findings about “superposition” and “polysemanticity” in neurons (Elhage et al., 2022; Kopf et al., 2025), showing that individual neurons represent an array of concepts rather than having a single function. Such findings may imply that feedforward layers of transformers similarly do not process syntax in an encapsulated way. Therefore, here we focus on attention heads, following the reasoning described in the previous paragraph.

To characterize the computations in attention heads that are specialized for particular syntactic dependencies, we tested whether they were penetrable to, or encapsulated from, a specific source of nonsyntactic information: semantic plausibility. We opted to test syntactic dependencies between pairs of words rather than larger syntactic trees of larger phrases because (1) dependency relations have been localized to specific attention heads in prior work, whereas entire syntactic trees might be too complex to be represented by single attention heads; and (2) this strategy allowed us a more targeted test of encapsulation versus penetrability, namely, whether the strength of attention toward a particular dependency between two words was influenced by the semantic plausibility of that very dependency (cf. testing whether the overall quality of a syntactic representation for an entire sentence is influenced by the “holistic” plausibility of that sentence, where the syntactic tree has many components and plausibility is influenced by many factors).

Whereas attention heads can, in principle, learn to assign weights between tokens based on any feature of the input, in practice, some heads learn to identify human‐interpretable features. For instance, some heads encode positional information (e.g., attend most strongly from each token to its previous token), some widely distribute attention over all tokens to create “bag of words” representations, some target specific parts of speech, and so on (Clark, Khandelwal, Levy, & Manning, 2019; Raganato & Tiedemann, 2018; Vig & Belinkov, 2019; Voita, Talbot, Moiseev, Sennrich, & Titov, 2019). Critically, some attention heads appear “specialized” for identifying specific types of syntactic dependencies between words: one head targets the nominal subject of a verb, another—the object of a preposition, and so on (Clark et al., 2019). Such heads assign more weight from a dependent to its head (or vice versa) than to any other token in a sentence. However, in these specialized attention heads, patterns of attention correspond to particular dependencies with less than perfect accuracy (e.g., only in 77% of instances, on average, across eight dependencies for which attention heads show the strongest specialization in Clark et al., 2019). Moreover, the attention that a head assigns to words in its preferred syntactic dependency can decrease in the presence of “syntactically interfering” material between those words (Ryu & Lewis, 2021). Given these results, we asked whether such syntax‐specialized attention heads were also influenced by factors other than syntax alone.

To answer our question, we first identified “syntax‐specialized” attention heads, each sensitive to a different syntactic dependency; then, we measured the attention weights allocated by each head to its preferred dependency, across minimal pairs of sentences where that dependency was either semantically plausible/likely or implausible/unlikely. If computations in these “syntax‐specialized” heads are encapsulated, then the strength of attention to the preferred dependency should not vary across the two types of sentences. If, however, those computations are penetrable to semantic information—as we predicted, based on parallels with the human mind—then attention weights for the preferred dependency should be lower in sentences where that dependency is implausible compared to plausible. Such a finding would provide strong evidence that syntactic processing in LLMs—like in humans—is penetrable to semantics.

As a secondary hypothesis, we predicted that, when the preferred syntactic dependency of an attention head is semantically implausible, some attention might be targeted toward other words that are more plausible candidates for that dependency in terms of their meaning, even if their syntactic position cannot participate in that dependency; such attention would be stronger compared to attention toward words at the same syntactic position that are semantically implausible candidates for the dependency and appear in sentences where the true syntactic dependency is plausible.

## 2 Methods

### 2.1 Model description

We analyzed three transformer‐based LLMs: the “base” sized BERT model (BERT‐base‐uncased) (110M parameters; Devlin, Chang, Lee, & Toutanova, 2019), the GPT‐2 small model (117M parameters; Radford et al., 2019), and Llama 2 in its 7B‐parameter version (Touvron et al., 2023). BERT has 12 layers, each with 12 associated attention heads; it is a bidirectional model (directing attention to both preceding and following words) trained on both masked word prediction (i.e., “filling in” a missing word in a sentence) and next sentence prediction. GPT‐2 small has the same dimensionality as BERT, but it is a unidirectional model (i.e., backward‐looking) trained on next word prediction. Llama 2 has 32 layers, each with 32 associated attention heads and, like GPT‐2 small, is unidirectional and trained on next word prediction. All LLMs were accessed via the Hugging Face Transformers library (Wolf et al., 2019).

### 2.2 Identifying “syntax‐specialized” attention heads

First, for each of 43 dependency types from the Stanford Dependencies (de Marneffe, MacCartney, & Manning, 2006), we searched for specialized attention heads that accurately encoded that dependency. Our search procedure is a modified version of the one from Clark et al. (2019), who identified such heads in BERT.

To identify these heads, we first annotated the development set (section 22) of the Penn Treebank 2 corpus (Clark et al., 2019; Marcus, Santorini, & Marcinkiewicz, 1993) with Stanford dependencies, using the Stanford CoreNLP toolbox (Manning et al., 2014). For each dependency and for each head, specialization was measured in two ways: (1) the percentage of instances of the dependency in the corpus for which the *head* token directed more attention to the *dependent* token than to any other token (denoted head→dep); and (2) the percentage of instances of the dependency in the corpus for which the *dependent* token directed more attention to the *head* token than to any other token (denoted dep→head).^5^ Some of the heads we identified exhibited specialization based on measure (1), and others—based on measure (2). Using both measures allows us to obtain a larger set of “syntax‐specialized” attention heads for our analyses. Furthermore, for our purposes, the critical feature of a “syntax‐specialized” attention head is that it identifies a relationship of a particular kind between two words, not that the direction of that relationship aligns with linguistic theory (and, for some dependencies, there are theoretical disagreements regarding which word constitutes the head).

For GPT‐2 small and Llama 2, we excluded sentences that required forward‐facing attention (e.g., measuring attention from a dependent to a head that comes later in the sentence), as those models are unidirectional (attend only to the current token and preceding context). For the “Indirect Object” dependency, too few instances existed in the original corpus (folder 22), so we used 203 sentences featuring this dependency from various folders of the Penn Treebank.

For GPT‐2 small, BERT, and Llama2, we identified multiple attention heads specialized for syntactic dependencies. Note that some dependencies in the corpus could be identified with some accuracy by merely attending to tokens at a fixed distance (e.g., the object of a preposition is often two tokens to the right of the preposition, as in “*up a tree*,” “*on the table*,” etc.). An attention head that is “syntax‐specialized,” rather than distance‐specialized, should have a specialization score higher than this fixed‐offset baseline (Clark et al., 2019). Therefore, for each dependency type, we found the most common distance between tokens in that dependency across the corpus and measured the percentage of instances exhibiting that distance. Attention heads were considered specialized only if their scores were at least 10 percentage points above the baseline (Ryu & Lewis, 2021). If more than five attention heads met this criterion, we analyzed the five heads with the highest specialization scores. To identify the specialized heads, for GPT‐2 small and BERT, we computed a set of 288 scores per dependency (12 layers × 12 heads × 2 measures). For Llama 2, the heads with the highest scores for each dependency were selected from a set of 2048 scores (32 layers × 32 heads × 2 measures) (see also note in Table 1).

**Table 1:** Heads specialized for different dependencies

BERT				
Relation	Head	Direction	Specialization score	Baseline score (distance)
Direct Object	7–9	dep→head	86.47	39.78 (−2)
Adverb Modifier	7–3	head→dep	51.97	41.35 (−1)
Nominal Subject	7–1	dep→head	60.89	46.46 (1)
Passive Nominal Subject	4–10	head→dep	62.45	43.87 (−2)
Object of Preposition	7–10	head→dep	81.95	34.67 (2)
Indirect Object	6–9	dep→head	77.07	46.34 (−1)

GPT‐2 small				
Relation	Head	Direction	Specialization score	Baseline score (distance)
Direct Object	2–8	dep→head	81.63	40.63 (−2)
Adverb Modifier	2–8	dep→head	65.24	52.14 (−1)
Nominal Subject	4–3	head→dep	66.87	48.66 (−1)
Passive Nominal Subject^a^	4–3	head→dep	73.30	44.20 (−2)
Object of Preposition	2–0	dep→head	76.55	34.62 (−2)

Llama 2				
Relation	Head	Direction	Specialization score	Baseline score (distance)
Direct Object	4–11	dep→head	74.63	40.63 (−2)
Nominal Subject	3–22	head→ dep	65.06	48.7 (−1)
Passive Nominal Subject^a^	4–30	head→dep	70.50	44.20 (−2)
Object of Preposition^a^	10–17	dep→head	67.00	34.60 (−2)

^a^ For these dependencies, the heads that originally obtained the highest specialization score attended to the dependency in the direction that is opposite to the one stated in the table. However, this specialization score was based on too few instances of the dependency in the corpus. Therefore, we replaced these heads with heads that showed the highest specialization score for the direction shown in the table, which had enough corpus instances. Note that, because GPT‐2 small and Llama 2 are unidirectional (i.e., do not attend to tokens to the right), instances of the dependency in one direction (e.g., from a head to a preceding dependent) are distinct from instances in the opposite direction (from a dependent to a preceding head).

For the main experiment, we chose six dependency types for which we identified specialized heads (Table 1) and for which we could feasibly create minimal sentence pairs, as described below. These dependencies are Direct Object, Adverb Modifier, Nominal Subject, Passive Nominal Subject, Object of a Preposition, and Indirect Object (see examples in Table 2). Our main analysis focused on the most syntax‐specialized head for each dependency in each LLM; see Supplementary Appendix D for the full set of specialized heads and Supplementary Appendix E for the distribution of syntax‐specialized attention heads in each model. Not all models were tested on all dependency types (see Section 2.3.1).

**Table 2:** Example stimuli: minimal pairs for each dependency (for BERT)

Dependency	Plausible (or likely)	Implausible (or unlikely)
Direct Object	The guide **showed** the visitor a **sculpture**.	The guide **showed** the sculpture a **visitor**.
Adverb Modifier	The song that was **played loudly** was later silenced.	The song that was **silenced loudly** was later played.
Nominal Subject	The **artist** by the window **painted** a landscape	The **window** by the artist **painted** a landscape.
Passive Nominal Subject	The **house** in the novel was **sold** by the agent.	The **house** in the novel was **read** by the agent.
Object of Preposition	It was the **shoreline** by the rocks that the waves crashed **against**.	It was the **transparency** by the rocks that the waves crashed **against**.
Indirect Object	The guide **showed** the **visitor** a sculpture.	The guide **showed** the **sculpture** a visitor.

*Note*. Words in bold constitute the critical dependency. Attention is directed from the underlined word to the other bolded word. Words in red are “lures.”

### 2.3 Main experiment

#### 2.3.1 Stimuli

For each dependency, we constructed 50 minimal pairs of sentences such that in one sentence the phrase contained within the dependency was semantically plausible in the broader context of the sentence, and in the other—semantically implausible (Table 2). For example, for the Nominal Subject dependency, a plausible sentence would read “*the artist by the window painted a landscape*,” and its implausible version would read “*The window by the artist painted a landscape*.” All sentences for a given dependency had identical structure, and each word comprised a single token in all three models. For all dependencies other than Object of a Preposition and Passive Nominal Subject Dependencies, plausibility was manipulated by swapping two words in the sentence that both had the same part of speech. For the Object of a Preposition, plausibility was manipulated by replacing the prepositional object with a different noun (i.e., each sentence in a pair had a noun that the other sentence did not). For the Passive Nominal Subject Dependency, only the main verb was manipulated within sentence pairs. The Direct Object and Indirect Object dependencies used identical stimuli that contained both dependency types: ditransitive, double‐object sentences. In a supplementary experiment (see Supplementary Material, Appendix A), we obtained ratings of our stimuli from human participants to validate the plausibility levels, and we also tested whether human plausibility judgments predict attention strength on the item level.

Many of our dependency types contained no temporary syntactic ambiguity. First, the sentence structures that we used for all dependency types did not have *global* syntactic ambiguities. Second, because BERT is a bidirectional model, even when the attention‐directing word was not the final word in the sentence, the full context of the sentence was available. While GTP2‐small and Llama 2 are unidirectional models, the dependent in the Direct Object and Object of a Preposition dependencies was the final word, so the full context of the sentence was available in these cases. In the Passive Nominal Subject dependency, the partial context prior to the critical words was unambiguous (“The house in the novel was sold”). In these cases, because the sentences are structurally unambiguous, there is in principle no need to rely on semantic information to parse the sentence's structure. Therefore, if LLMs do leverage semantic information to process syntactic dependencies even when it is not required, this would provide strong evidence that their representations of syntax are penetrable to semantics.

BERT was tested on all six dependencies. For unidirectional models, we did not analyze the Indirect Object dependency, because (1) in incremental processing, the noun is ambiguous between the direct and indirect object (namely, when encountering “*the guide showed the visitor…*,” the second noun could be either an indirect object to be followed by, e.g., “*a sculpture*,” or a direct object followed by, e.g., “*to the manager*”). Additionally, for GPT‐2, the same head was identified as specialized for both the Indirect Object and Direct Object (perhaps reflecting the ambiguity above).^6^ Therefore, GPT‐2 was tested on five dependencies, excluding Indirect Object. Llama 2 was tested on three dependencies for which specialized heads could be identified: Direct Object, Passive Nominal Subject, and Object of a Preposition (again, excluding Indirect Object).

Across LLMs, there were slight differences in the stimuli due to differences in tokenization: if a critical word was processed as a single token by one LLM but broken into several tokens in another, it was replaced for the latter model by an appropriate single‐token word. In addition, sentences for the Object of a Preposition dependency had one form for BERT and another for GPT‐2 small and Llama 2: in BERT, the preposition came several tokens after the prepositional object. But because GPT‐2 small and Llama 2 are unidirectional and the specialized head we found attended from the dependent—that is, the object—to the preposition, this preposition had to occur before the object. Thus, sentences differed in structure across the LLMs for the Object of a Preposition dependency, but they were roughly matched in overall semantic content (BERT: “*It was the shoreline by the rocks that the waves crashed against*”; GPT‐2: “*The waves crashed against the coast's strongly built dock*.”) Aside from tokenization‐related changes, minor semantic modifications were made to ensure naturalness given the syntactic differences for the Object of a Preposition stimuli used for BERT versus those for GPT‐2 and Llama 2.

To test our secondary hypothesis, each sentence contained a “lure” word that was not part of the critical dependency but, in the implausible sentences, was a semantically plausible target for that dependency (Table 2). For instance, for the Nominal Subject dependency, the implausible sentence “*The window by the artist painted the landscape*” contains the word “artist,” which is not the nominal subject of the verb, but it is a good semantic candidate for such a dependency (because an artist is an animate entity that is likely to paint something).

#### 2.3.2 Analysis

For a given LLM and dependency type, we fed each sentence separately to the model and extracted attention patterns from the head of interest. We analyzed the attention weights between critical words in our stimuli, contrasting the plausible and implausible conditions. Because attention weights are bounded in [0,1], we applied the logit‐transformation to them prior to statistical analysis. Data for each dependency type and LLM were analyzed separately. Statistical results were Bonferroni corrected for multiple comparisons across dependency types, with each pair of LLM and statistical model type (simple vs. full model) being treated as a family of comparisons.

To test our main hypothesis, we first modeled attention strength between words in the critical dependency (either from the head to the dependent, or from the dependent to the head, depending on the identified attention head; see Table 1). The model included two fixed effects: plausibility (plausible vs. implausible) and, as a covariate, log‐frequency for the word in the critical dependency that differed between two sentences in a pair (from the SUBTLEX_US_ corpus; Brysbaert & New, 2009) (for evidence that word frequency affects LLM performance, see, e.g., Kauf et al., 2023). In other words, the two sentences in each pair were treated as two observations, each contributing its own attention score (for the critical dependency) and log‐frequency (for its critical word). The covariate of word frequency was included to capture two effects: differences between different pairs of stimuli, as well as differences between two sentences in a pair (because the frequency of the critical word that differed between the plausible and implausible versions was not matched). The model also included a random intercept by sentence pair, unless it did not converge, in which case, it was reduced to a fixed‐effects model. We predicted that, compared to plausible sentences, implausible sentences would show lower attention to the syntactically correct target in the critical dependency.

Second, to determine whether semantic plausibility predicts attention strength even when controlling for the statistical dependencies between the critical words and their general semantic similarity, we modeled attention strength using additional control variables. Specifically, we ran a linear mixed‐effects model identical to the one above, but with pointwise mutual information (PMI) (measuring statistical co‐occurrence) and cosine similarity (measuring semantic relatedness) between critical word pairs as additional fixed‐effects. We computed PMI estimates using a Word2Vec model trained on the English Wikipedia corpus downloaded in December 2021 (Hengchen, 2022), using an identical procedure to the one used in Hoover, Du, Sordoni, and O'Donnell (2021). Cosine similarity scores were computed using word vectors from the same Word2Vec model. Third, because LLMs are trained on a word prediction objective, we explored the relationship between plausibility and predictability in a supplementary experiment that tested the effects of plausibility when controlling for surprisal (see Supplementary Material, Appendix B, and the Discussion section).

In a secondary analysis, we analyzed attention strength involving the “lure” word (Table 3). Lure words always had the same part of speech as one of the words in the critical dependency and were semantically *better* candidates for the dependency in implausible (vs. plausible) sentences. For some dependencies, the lure word received attention from the head/dependent of the critical dependency; for instance, for the Nominal Subject dependency in GPT‐2, the lure word was the second noun (“window”) in the plausible/implausible pair “*The* ***artist*** *by the window painted a landscape*”/ “*The* ***window*** *by the artist painted a landscape*”). In this case, “window” was the lure word because it has the same part of speech as the first noun and is a more plausible candidate for the subject of “*painted*” in terms of its semantic meaning. For this dependency, the main verb (“painted”) was the attention‐directing word for both the critical and secondary analyses, because our corpus analysis found the “nominal‐subject” head to be specialized in the head (i.e., main verb) → dependent direction. In other cases, the lure word directed attention, and the head or dependent from the original dependency received attention from the lure. For instance, for a plausible/implausible pair for the Direct Object dependency (e.g., “*The guide* ***showed*** *the visitor a sculpture* **.”** */* “*The guide* ***showed*** *the sculpture a visitor* **.”**), the indirect object was selected as the lure word, directing attention to the main verb. This is analogous to the method used for the critical analysis of the same dependency, in which we analyzed attention directed from the final noun (i.e., the direct object) to the main verb. All analyses used the same two linear, mixed‐effects models with the same structures (and multiple‐comparison correction) as for the main analysis. We predicted that, compared to plausible sentences, implausible sentences would show higher attention to/from the syntactically incorrect lure.

**Table 3:** Direction of attention analyzed in the secondary (“lure” word) analysis

Dependency	BERT	GPT‐2	Llama 2
Direct Object	Lure→Head	Lure→Head	Lure→Head
Adverb Modifier	Lure→Dep	Lure→Head	N/A
Nominal Subject	Lure→Head	Head→Lure	N/A
Passive Nominal Subject	Head→Lure	Head→Lure	Head→Lure
Object of Preposition	Head→Lure	N/A	N/A
Indirect Object	Lure→Head	N/A	N/A

*Note*. “Head” and “dep” refer to words that are part of the syntactically correct dependency. In cells for which the lure word analysis was not conducted, N/A is shown.

We further wanted to assess whether, in implausible sentences, an increase in attention was specific to the lure word (rather than reflecting diffused increased attention across all words that are not part of the critical dependency). To this end, we conducted an additional analysis comparing attention to the lure word versus a control word.^7^ Specifically, in cases where the head or dependent directed (rather than received) attention in the lure analyses, attention may generally increase for words outside of the critical dependency in implausible sentences. For example, in the Nominal Subject dependency for GPT‐2, if attention from the main verb “painted” to the subject “window” decreases in the implausible sentence “*The* ***window*** *by the artist painted a landscape*,” attention might be evenly reallocated across all words preceding “painted” rather than selectively allocated to the lure word. However, if an attention head treats the lure word as a potential candidate for the dependency, attention should increase specifically for the lure word but not for a control word with a different part of speech.

For GPT‐2 and Llama 2, we tested the Nominal Subject and Passive Nominal Subject dependencies, and for BERT, we tested the Passive Nominal Subject dependency, which met the above condition (head/dependent directing rather than receiving attention). The control word had to have (1) a minimum distance of two dependencies from the attention‐directing word in the dependency graph, and (2) a minimum distance of two dependencies from the syntactically correct target word that the attention‐directing word normally attends to (i.e., in plausible sentences). For instance, for the Passive Nominal Subject dependency in GPT‐2 (e.g., Plausible: “The house in the novel was sold by the agent.”; Implausible: “The house in the novel was read by the agent.”), we analyzed attention from the main verb (Plausible: “sold”; Implausible: “read”) to the lure word “novel” and to the control word “in,” which met the two criteria above. We fit a linear mixed‐effects model with a fixed‐effect interaction between plausibility (plausible vs. implausible) and word type (lure vs. control), with log word frequency (from the SUBTLEXUS corpus) as a continuous covariate, and a random intercept by sentence pair. Following the interaction test, we conducted Bonferroni‐corrected pairwise comparisons to examine the simple effects of plausibility on attention to lure and control words. All pairwise comparisons were performed using the *emmeans* package in R (Lenth, 2017).

## 3 Data availability

Stimuli, data, and code for replicating the results of this experiment are available through the following OSF link. Upon publication, all materials will be publicly accessible via GitHub.

## 4 Results

Our main question was whether attention heads that appear to be “syntax‐specialized” for particular dependencies modulated their attention to their preferred dependency based on its semantic plausibility. Consistent with our prediction, in most cases, attention strength between words in the preferred dependency was higher in plausible versus implausible sentences. We also analyzed attention directed to/from “lure” words, which were syntactically incorrect targets for the dependency but, in the implausible sentences, were semantically plausible candidates for that dependency. In a subset of cases, attention weights directed to/from lures were higher in the implausible sentences than in the plausible sentences (where these lures were not semantically plausible candidates for the critical dependency). For each LLM and dependency type, results for the single most specialized head are reported here; results for the other specialized heads tested are reported in Supplementary Appendix F. Reported *p‐*values are uncorrected but were evaluated against Bonferroni‐corrected thresholds (α divided by the number of comparisons), with each LLM × statistical model type (full vs. simple) treated as a separate family of comparisons.

Our identification of putatively syntax‐specialized attention heads used sentences from the Penn Treebank, which are likely mostly plausible. Because this method identifies heads with particularly high attention weights for dependencies whose content is plausible, it may bias our search toward heads that then show the critical “plausible > implausible” effect in our analyses. For example, our identification method might have found heads that are sensitive to semantic relatedness in addition to (or, perhaps, more strongly than to) syntactic dependencies per se.

To address this concern, in a supplementary experiment, we identified attention heads with a different method: using a newly constructed stimulus set with an equal number of plausible and implausible sentences. Then, we tested the penetrability of these heads to plausibility information using the same stimulus set reported here (which is independent from the set used to identify the heads). The results are reported in Supplementary Appendix G and are qualitatively similar to our main analyses.

### 4.1 BERT

#### 4.1.1 Main analysis

**Fig. 1:**
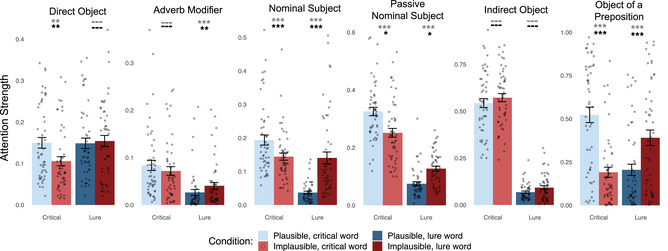
BERT attention strengths between words in a critical dependency, extracted from “syntax‐specialized” attention heads. Each panel displays data for a different syntactic dependency. Dots correspond to individual sentences, with bars showing averages, and error bars—standard errors. Data compare plausible (blue) and implausible (red) sentences; bright bars show attention for the critical, syntactically correct dependency, and dark bars show attention to/from semantic lures. Black stars indicate significance for the full model, and gray stars indicate the significance for the base model. The significance of Bonferroni‐corrected *p*‐values is reflected above the plots: “—” for nonsignificant, * for *p*<.05; ** for *p*< .01; and *** for *p*<.001.

Results are presented in Fig. 1. For the base model containing only Plausibility and Log Word Frequency as fixed‐effects, attention weights between critical words were significantly higher in the plausible condition than in the implausible condition for four out of six dependencies: Direct Object (*b* = 0.54, *SE* = 0.15, *t*_(49.60)_ = 3.64, *p* = .00033), Nominal Subject (*b* = 0.41, *SE* = 0.086, *t*_(48.37)_ = 4.73, *p*<10^−4^), Passive Nominal Subject (*b* = 0.40, *SE* = 0.10, *t*_(48.50)_ = 3.91, *p* = .00015), Object of a Preposition (*b* = 2.26, *SE* = 0.26, *t*_(48.44)_ = 8.54, *p*<10^−4^). The effect was nonsignificant for Adverb Modifier (*b* = 0.30, *SE* = 0.13, *t*_(48.44)_ = 2.30, *p* = .013) (α = 0.05/6), Indirect Object (*b* = −0.16, *SE* = 0.11, *t*_(49.44)_ = −1.37, *p* = .18), and for a different syntactic construction featuring the Object of a Preposition dependency (Supplementary Appendix C).

For the full model containing Plausibility, Log Word Frequency, PMI, and Cosine Similarity, attention strength between critical words was significantly higher in the plausible condition than in the implausible condition for four out of six dependencies: Direct Object (*b* = 0.47, *SE* = 0.15, *t*_(53.61)_ = 3.12, *p* = .0015), Nominal Subject (*b* = 0.43, *SE* = 0.087, *t*_(49.08)_ = 4.916, *p*<10^−4^), Passive Nominal Subject (*b* = 0.44, *SE* = 0.17, *t*_(72.03)_ = 2.65, *p* = .0049), and Object of a Preposition (*b* = 2.12, *SE* = 0.36, *t*_(57.75)_ = 5.83, *p*<10^−4^); this difference was not significant for the Adverb Modifier dependency (*b* = 0.17, *SE* = 0.15, *t*_(52.51)_ = 1.15, *p* = .13). Additionally, there was a nonsignificant effect opposite of what we predicted for the Indirect Object dependency (*b* = −0.26, *SE* = 0.12, *t*_(52.72)_ = −2.18, *p* = .017).

#### 4.1.2 Lure analysis

For the base model, attention weights directed to/from lure words showed the opposite pattern to the results of the main analysis (as predicted) and were significantly higher in the implausible condition for four out of six dependencies: Adverb Modifier (*b* = −0.83, *SE* = 0.17, *t*_(48.77)_ = −4.84, *p*<10^−4^), Nominal Subject (*b* = −1.54, *SE* = 0.18, *t*_(97)_ = −8.74, *p*<10^−4^), Passive Nominal Subject (*b* = −0.66, *SE* = 0.11, *t*_(48.60)_ = −6.02, *p*<10^−4^), and Object of a Preposition (*b* = −1.26, *SE* = 0.23, t_(49.00)_ = −5.60, *p*<10^−4^). This pattern did not hold for the Direct Object (*b* = −0.076, *SE* = 0.10, *t*_(48.31)_ = −0.74, *p* = .23) or Indirect Object (*b* = −0.18, *SE* = 0.15, *t*_(49.22)_ = −1.23, *p* = .112).

For the full model, attention weights directed to/from lure words were significantly higher in the implausible condition for four out of six dependencies: Adverb Modifier (*b* = −0.65, *SE* = 0.19, *t*_(53.64)_ = −3.50, *p* = .00047), Nominal Subject (*b* = −1.60, *SE* = 0.17, *t*_(95)_ = −9.08, *p*<10^−4^), Passive Nominal Subject (*b* = −0.51, *SE* = 0.18, *t*_(68.01)_ = −2.88, *p* = .0027), and Object of a Preposition (*b* = −1.26, *SE* = 0.23, *t*_(49.00)_ = −5.60, *p*<10^−4^). This pattern did not hold for the Direct Object (*b* = −0.076, *SE* = 0.11, *t*_(50.85)_ = −0.672, *p* = .25) or Indirect Object (*b* = −0.25, *SE* = 0.15, *t*_(50.31)_ = −1.61, *p* = .057).

### 4.2 GPT‐2 small

#### 4.2.1 Main analysis

**Fig. 2:**
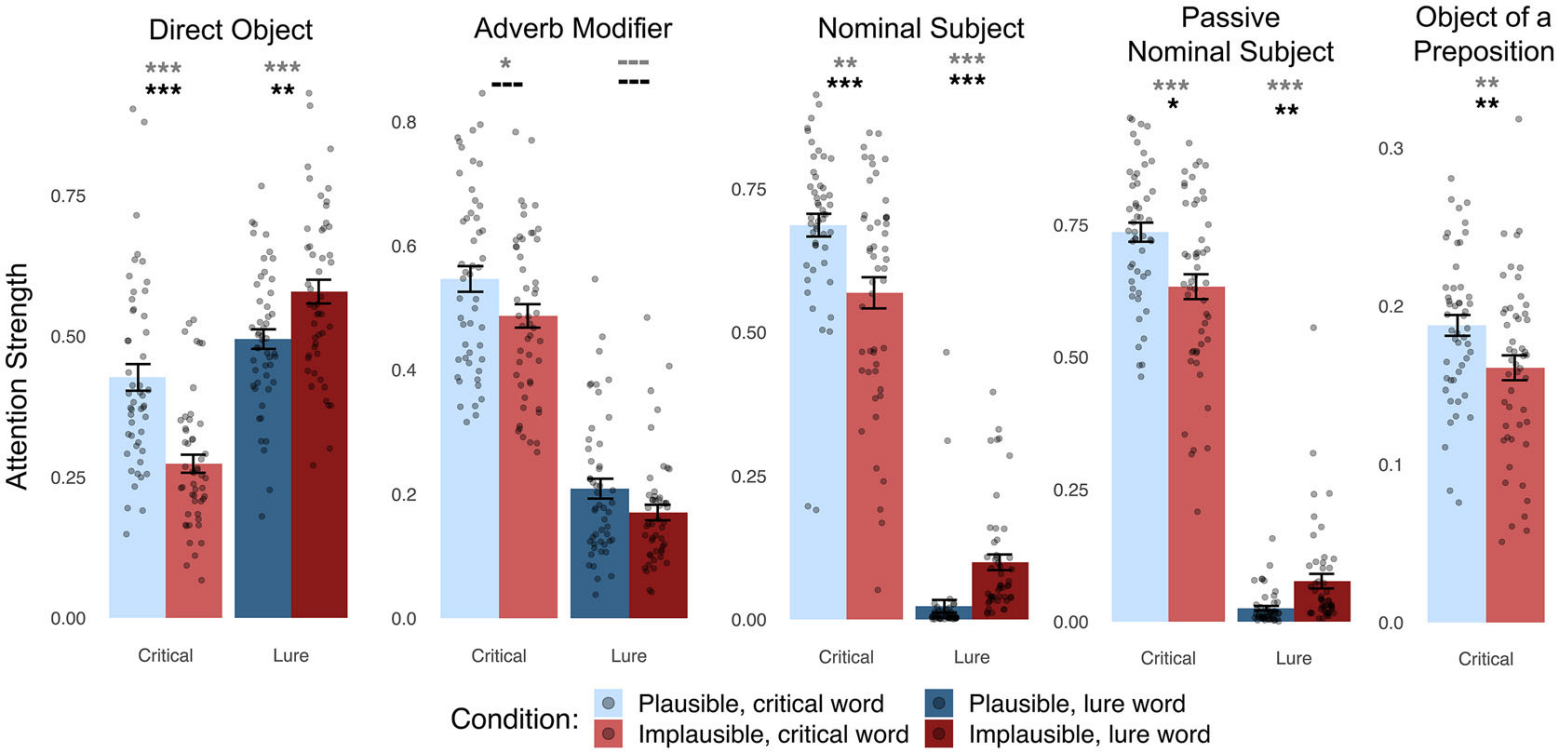
GPT‐2 small attention strengths between words in a critical dependency, extracted from “syntax‐specialized” attention heads. Conventions are the same as Fig. 1.

Results are presented in Fig. 2. For the base model containing only Plausibility and Log Word Frequency as fixed‐effects, attention strength between critical words was significantly higher in the plausible condition than in the implausible condition for all five dependencies: Direct Object (*b* = 0.80, *SE* = 0.13, *t*_(49.86)_ = 6.33, *p*<10^−4^), Adverb Modifier (*b* = 0.26, *SE* = 0.10, *t*_(48.02)_ = 2.49, *p* = .0082), Nominal Subject (*b* = 0.55, *SE* = 0.15, *t*_(48.56)_ = 3.74, *p* = .00024), Passive Nominal Subject (*b* = 0.54, *SE* = 0.12, *t*_(48.18)_ = 4.62, *p*<10^−4^), and Object of a Preposition (*b* = 0.23, *SE* = 0.062, *t*_(46.49)_ = 3.73, *p* = .00026).

For the full model containing Plausibility, Log Word Frequency, PMI, and Cosine Similarity, attention strength between critical words was significantly higher in the plausible condition than in the implausible condition for three out of five dependencies: Direct Object (*b* = 0.70, *SE* = 0.13, *t*_(53.62)_ = 5.42, *p*<10^−4^), Nominal Subject (*b* = 0.57, *SE* = 0.15, *t*_(48.76)_ = 3.81, *p* = .00020), and Passive Nominal Subject (*b* = 0.59, *SE* = 0.21, *t*_(62.47)_ = 2.86, *p* = .0029). However, differences were nonsignificant for the Adverb Modifier (*b* = 0.19, *SE* = 0.11, *t*_(53.19)_ = 1.69, *p* = .048) and Object of a Preposition (*b* = 0.12, *SE* = 0.075, *t*_(60.54)_ = 1.65, *p* = .052) dependencies.

#### 4.2.2 Lure analysis

Results are presented in Fig. 2. For the base model, attention weights directed to/from lure words showed the opposite pattern to the main analysis (as predicted) and were significantly higher in the implausible condition than in the plausible condition for three out of four dependencies tested: Direct Object (*b* = −0.45, *SE* = 0.11, *t*_(49.71)_ = −4.01, *p* = .00010), Nominal Subject (*b* = −2.55, *SE* = 0.22, *t*_(48.62)_ = −11.60, *p*<10^−4^), and Passive Nominal Subject (*b* = −1.22, *SE* = 0.19, *t*_(48.23)_ = −6.37, *p*<10^−4^). This difference was nonsignificant for the Adverb Modifier (*b* = 0.20, *SE* = 0.11, *t*_(48.71)_ = 1.91, *p* = .031). We did not analyze attention to/from the lure word for the Object of a Preposition dependency because the tokens leading up to, and including, the lure word were identical between the plausible and implausible conditions (and, therefore, attention weights from the lure to the preposition were identical).

For the full model, attention weights directed to/from lure words were significantly higher in the implausible condition for all four dependencies tested: Direct Object (*b* = −0.35, *SE* = 0.11, *t*_(53.09)_ = −3.08, *p* = .0016), Adverb Modifier (*b* = 0.30, *SE* = 0.12, *t*_(56.39)_ = 2.36, *p* = .011), Nominal Subject (*b* = −2.55, *SE* = 0.22, *t*_(48.22)_ = −11.47, *p*<10^−4^), and Passive Nominal Subject (*b* = −0.96, *SE* = 0.30, *t*_(66.84)_ = −3.16, *p* = .0012).

### 4.3 Llama 2

#### 4.3.1 Main analysis

**Fig. 3:**
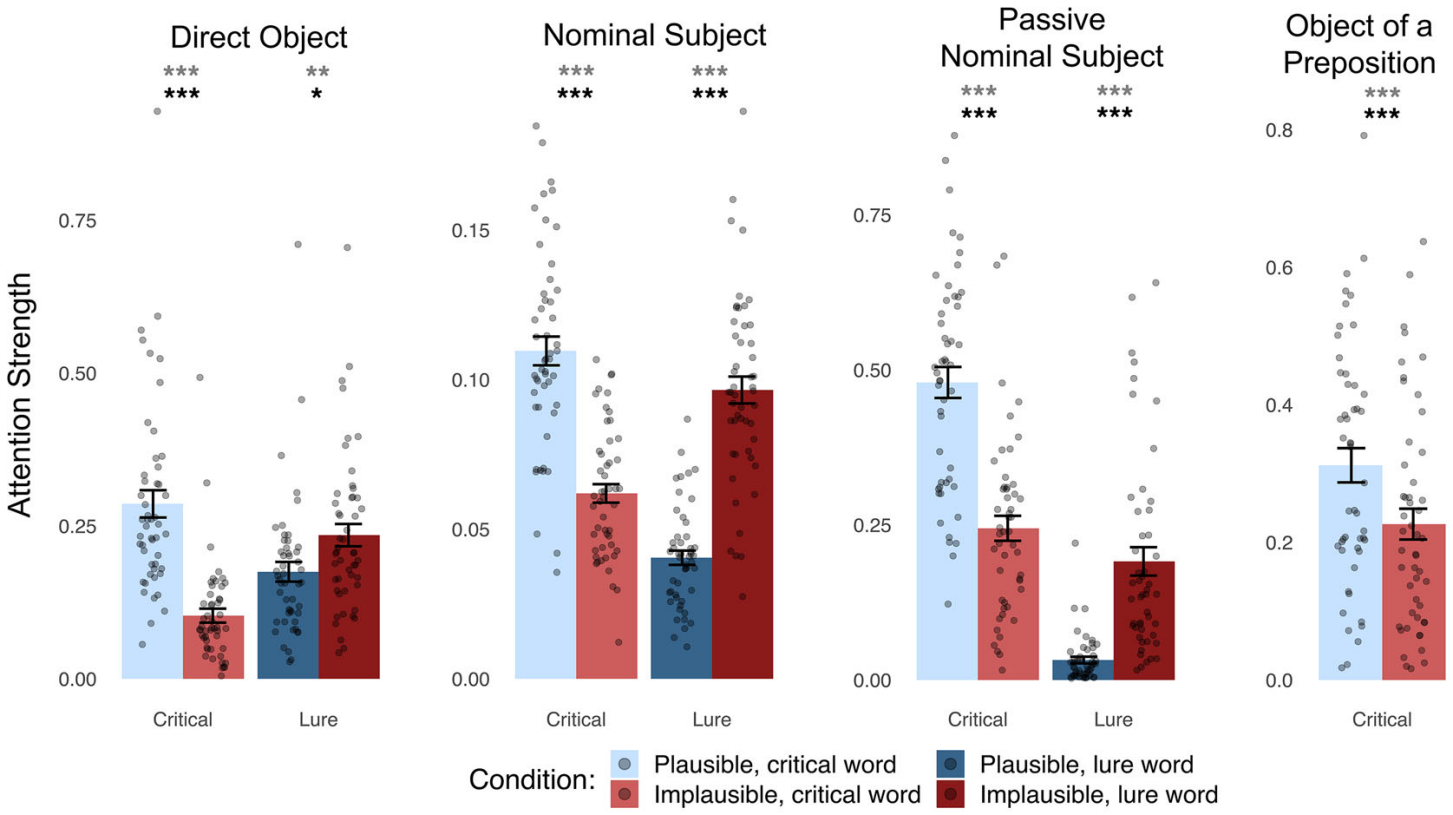
Llama 2 attention strengths between words in a critical dependency, extracted from “syntax‐specialized” attention heads. Conventions are the same as Fig. 1.

Results are presented in Fig. 3. For the base model, attention strength between critical words was significantly higher in the plausible condition than in the implausible condition for all four dependencies tested: Direct Object (*b* = 1.41, *SE* = 0.17, *t*_(97)_ = 8.24, *p*<10^−4^), Nominal Subject (*b* = 0.64, *SE* = 0.06, *t*_(47.57)_ = 10.62, *p*<10^−4^), Passive Nominal Subject (*b* = 1.23, *SE* = 0.15, *t*_(50.12)_ = 8.39, *p*<10^−4^), and Object of a Preposition (*b* = 0.53, *SE* = 0.12, *t*_(48.24)_ = 4.49, *p*<10^−4^). We did not test the Adverb Modifier dependency because the head that was most specialized for this dependency had accuracy that was not sufficiently higher than the fixed‐distance baseline (63.97% vs. 61.74%). For the Nominal Subject dependency, the most specialized head was specialized in the dependent → head direction (i.e., the subject had to attend back toward the verb, e.g., “‘*The markets showed a steady decrease*’, *said the reporter.*”); this direction severely limited the semantic contexts in which stimuli could be created, making it hard to create pairs of plausible and implausible sentences for the Nominal Subject dependency. Thus, for this dependency, we tested the head that was most specialized in the head → dependent direction (accuracy score: 65.1%; fixed‐distance baseline accuracy: 48.7%) using sentences with the same structure as the one shown in Table 1.

For the full model containing Plausibility, Log Word Frequency, PMI, and Cosine Similarity, attention strength between critical words was significantly higher in the plausible condition than in the implausible condition for all four dependencies tested: Direct Object (*b* = 1.44, *SE* = 0.19, *t*_(95)_ = , *p*<10^−4^), Nominal Subject (*b* = 0.67, *SE* = 0.062, *t*_(48.68)_ = 10.78, *p*<10^−4^), Passive Nominal Subject (*b* = 1.01, *SE* = 0.21, *t*_(66.39)_ = 4.87, *p*<10^−4^), and Object of a Preposition (*b* = 0.57, *SE* = 0.15, *t*_(54.89)_ = 3.74, *p* = .00022).

#### 4.3.2 Lure analysis

Results are presented in Fig. 3. For the base model, attention weights directed to/from lure words showed the opposite pattern to the main analysis (as predicted) and were significantly higher in the implausible condition than in the plausible condition for all three dependencies tested: Direct Object (*b* = −0.38, *SE* = 0.13, *t*_(48.78)_ = −2.99, *p* = .0022), Nominal Subject (*b* = −0.96, *SE* = 0.055, *t*_(48.59)_ = −17.39, *p*<10^−4^), and Passive Nominal Subject (*b* = −2.24, *SE* = 0.22, *t*_(97)_ = −10.17, *p*<10^−4^). Like in GPT‐2 small, no lure analysis was conducted for the Object of a Preposition dependency.

For the full model, attention weights directed to/from lure words showed the opposite pattern and were significantly higher in the implausible condition for all three dependencies tested: Direct Object (*b* = −0.38, *SE* = 0.15, *t*_(62.23)_ = −2.54, *p* = .0068), Nominal Subject (*b* = −0.96, *SE* = 0.06, *t*_(48.44)_ = −16.63, *p*<10^−4^), and Passive Nominal Subject (*b* = −2.13, *SE* = 0.26, *t*_(62.12)_ = −8.26, *p*<10^−4^).

### 4.4 Testing the specificity of attention increases for the lure word

For some syntactic dependencies, attention involving the “lure” word was higher in implausible versus plausible sentences. To test whether this increase in attention was specific to the lure word, we tested the interaction between plausibility (plausible vs. implausible) and word type (lure vs. control). In line with our prediction, in BERT, we found a significant interaction of plausibility (plausible vs. implausible) and word type (lure or control word) (*b* = −0.56, *SE* = 0.16, *t*_(145.63)_ = −3.56, *p* = 5.1×10^−4^) for the Passive Nominal Subject dependency, which was the only dependency tested. Simple effects tests indicated that the estimated marginal mean for attention strength was greater in implausible than plausible sentences for lure words (*ΔM* = 0.66, *SE* = 0.11, *t*_(146)_ = 5.90, *p* < 10^−4^), but no such difference was observed for control words (*ΔM* = 0.096, *SE* = 0.11, *t*_(146)_ = 0.86, *p* = .39).

For GPT‐2, we found a significant interaction of plausibility and word type for both tested dependency types (Nominal Subject: *b* = −2.75 *SE* = 0.26, *t*_(146.18)_ = −10.37, *p* < 2×10^−16^; Passive Nominal Subject: *b* = −1.00, *SE* = 0.25, *t*_(146.37)_ = −3.95, *p* < 1.21×10^−4^). Simple effects tests confirmed that attention strength was greater for implausible than plausible sentences for lure words (Nominal Subject: *ΔM* = 2.55, *SE* = 0.19, *t*_(146)_ = 13.63, *p* < 10^−4^; Passive Nominal Subject: *ΔM* = 1.21, *SE* = 0.18, *t*_(146)_ = 6.80, *p* < 10^−4^), whereas attention to control words did not differ by plausibility (Nominal Subject: *ΔM* = −0.19, *SE* = 0.19, *t*_(146)_ = −1.04, *p* = .30; Passive Nominal Subject: *ΔM* = 0.22, *SE* = 0.18, *t*_(146)_ = 1.21, *p* = .23).

For Llama 2, we found a significant interaction of plausibility and word type for both dependencies (Nominal Subject: *b* = −1.00, *SE* = 0.11, *t*_(145.87)_ = −9.45, *p* < 2×10^−16^; Passive Nominal Subject: *b* = −2.46, *SE* = 0.26, *t*_(195)_ = −9.58, *p* < 2×10^−16^). Simple effects tests confirmed that attention strength was stronger in implausible than plausible sentences for lure words (Nominal Subject: *ΔM* = 0.96, *SE* = 0.075, *t*_(146)_ = 12.82, *p* < 10^−4^; Passive Nominal Subject: *ΔM* = 2.18, *SE* = 0.18, *t*_(195)_ = 11.97, *p* < 10^−4^), while there were no significant differences for control words (Nominal Subject: *ΔM* = −0.040, *SE* = 0.075, *t*_(146)_ = −0.53, *p* = .60; Passive Nominal Subject: *ΔM* = −0.28, *SE* = 0.18, *t*_(195)_ = −1.53, *p* = .13). For the Passive Nominal Subject analysis in Llama 2, the random intercept was removed because the mixed‐effects model failed to converge.

## 5 Discussion

We studied the computations of “syntax‐specialized” attention heads in three LLMs and found that the strength of attention that they assigned to their preferred dependency was influenced by semantic plausibility: attention was lower when the dependency was implausible (vs. plausible). For our base models, this pattern was observed in all cases but two (the Adverb Modifier and Indirect Object dependencies in BERT). For our full models, this pattern was observed in all cases but four (the Adverb Modifier and Indirect Object dependencies in BERT, and the Adverb Modifier and Object of a Preposition dependencies in GPT‐2). Moreover, in some cases, for implausible (vs. plausible) sentences, we found increased attention between words that, syntactically, do not constitute the preferred dependency, but are nonetheless semantically plausible candidates for that dependency. These findings demonstrate that the syntactic computations in these attention heads are penetrable to (at least some aspects of) semantics: they can be modulated by plausibility information. Such penetrability, or nonencapsulation, is functionally similar to the human language processing system, in which syntactic parsing is penetrable to semantics from the earliest moments of processing. Therefore, our findings lend further support to the use of LLMs as plausible neurocognitive models of syntactic processing.

This study presents a strong test for the encapsulation of syntactic computations in LLMs. First, we tested attention heads that appear to be strongly specialized for specific syntactic dependencies (although they do not identify their preferred dependency in 100% of the cases; Table 1). Given that attention heads link words in a sentence to one another (via attention weights), they are an a priori likely site for “purely” syntactic computations, if such computations existed. Of course, we do not exclude the possibility that syntactic encapsulation might exist elsewhere in LLMs (e.g., in other attention heads, in the feedforward layers, or for other types of syntactic information). We did not study feedforward output and residual stream embeddings because they contain information conveyed from all attention heads in the preceding attention block, and, therefore, likely encode many linguistic features in an entangled manner (as reviewed in the Introduction). While it is possible that LLMs represent syntax in an encapsulated fashion within a subspace of the residual stream embeddings (e.g., Geiger, Wu, Potts, Icard, & Goodman, 2024), we leave investigations of such representations to future studies. Interestingly, studies that probe syntactic information in such embeddings (e.g., Manning et al., 2020; Diego‐Simón et al., 2024) often do not evaluate the effect of meaning, so it remains possible that even those representations are influenced by semantic information. Such an assumption has implicitly or explicitly motivated other work (e.g., Huang, Huang, & Chang, 2021; Kumar et al., 2024; Bhalla et al., 2025). It may be particularly interesting to test whether mixture‐of‐experts models (e.g., Jiang et al., 2024)—where tokens can be routed to a number of specialized feedforward neural networks in parallel—process syntax in an encapsulated fashion. Our findings provide support for syntactic penetrability and against syntactic encapsulation in the attention heads of LLMs. However, LLMs have various kinds of syntactic representations across different model components and circuits, and characterizing these representations is an ongoing effort in the LLM interpretability literature. Hence, we emphasize that future work should investigate whether other syntactic representations in LLMs are encapsulated. Still, *if* there is syntactic encapsulation anywhere within LLMs, then the attention heads we identified are arguably the best a priori candidates for carrying out such computations. We provide compelling evidence that their computations are instead penetrable to semantic information.

Second, many of our stimuli contained no temporary syntactic ambiguity. For example, BERT is a bidirectional LLM, that is, it has access to all words in a sentence, and all six dependencies we tested used materials whose overall structure is unambiguous. Whereas GTP2‐small and Llama 2 are unidirectional, that is, only left‐looking, we emphasize that the dependent in the Direct Object and Object of a Preposition dependencies was the last word (so the entire structure was available at that point); and in the Passive Nominal Subject dependency, the partial context prior to the critical words was unambiguous (“*the house in the novel was sold*”).^8^ Because there is no need for syntactic disambiguation, there is, in principle, no need to rely on semantic information to parse the structure of the sentence. This lack of ambiguity contrasts with studies of syntactic encapsulation in humans, where the sentence stimuli are often temporarily ambiguous and thus reliance on nonsyntactic information confers an advantage during processing (Trueswell et al., 1994; Tanenhaus et al., 1995; but see Sikos, Duffield, & Kim, 2016). And yet, despite the sufficiency of the syntactic cues for determining sentence structure in our stimuli, attention heads were still influenced by semantic information. This suggests that penetrability is not optionally triggered when needed but is rather inherent to the functionality of these attention heads. In some cases, semantic information might even override syntactic information, leading attention heads to incorrectly represent dependencies. For instance, in implausible sentences, attention heads specialized for the Direct Object dependency in all three LLMs (descriptively) assign more attention to the lure than to the syntactically correct direct object (compare the two red bars in Figs. 1−3).

Our approach compared attention strength between minimal pairs, without searching for an absolute threshold of attention that would separate all plausible sentences from all implausible sentences. This approach is similar to many studies that evaluate transformer models’ syntactic knowledge via the probabilities they assign to sentences, and compare such probabilities between minimal pairs instead of searching for a probability threshold that signals “grammaticality.” The reasoning behind this approach is described in Hu, Wilcox, Song, Mahowald, and Levy (2026). Sentences vary along many dimensions, and such variation can “nudge” attention strength. As one example, consider sentence length. In unimodal Transformers like GPT2, attention across all previous words (and the current word) must sum to 1. If the context before the current word is extremely long, some attention weights (however, low) would be assigned to those many words, which would effectively “steal” weight from the attention to the critical dependency. Thus, on average, attention to the critical dependency will be lower for sentences with longer versus shorter contexts. More generally, the fact that a variety of factors influence attention strength is evident from Table 1: the “specialization scores” of the attention heads we study are not 100%, meaning that they do not always assign the most attention to the critical dependency. They are not idealized feature detectors, but rather carry out fuzzy computations that are sensitive to many variables.

Given the variety of differences between sentences in general, one should not expect attention to a plausible dependency to always be higher than attention to an implausible dependency. Indeed, we found considerable overlap in attention strength between our sets of plausible and implausible sentences (Figs. 1−3). This is a feature, not a bug: the minimal pairs approach allows us to isolate specific variables that influence attention strength (just as in studies that isolate the effect of grammaticality on sentence probability). Here, we attempted to isolate plausibility. The reasoning behind the minimal pairs approach aligns with usage‐based theories of human language processing and, in our case, with a pervasive integration of semantic information into syntactic processes.

In a supplementary experiment in GPT‐2 small (see Supplementary Material, Appendix B), we tested whether the observed effects of plausibility could be accounted for by predictability, given that a semantically plausible syntactic dependency is also likely to be encountered more frequently (and thus be more predictable) than an implausible dependency during LLM training. We analyzed attention in sentences where the critical dependencies were plausible but surprising. We found that in some cases, the effect of plausibility on attention strength could not be fully explained by the surprisal of (a critical word in) that dependency; however, the plausibility effects were somewhat attenuated when controlling for surprisal. This result is expected, given that LLMs learn about plausibility via a word prediction objective. Furthermore, our analyses that included PMI as a covariate (i.e., the full model analyses) provide another predictability control. We found that many attention heads exhibit significant plausibility effects even when controlling for PMI and semantic relatedness.

Plausibility and predictability are distinct and can diverge in numerous ways. A sentence could be highly plausible but have low predictability due to, for example, a low‐frequency syntactic structure (e.g., garden‐path sentences can be perfectly plausible but elicit high surprisal at the disambiguating region; Ferreira & Henderson, 1991). A highly plausible sentence could also contain a low‐frequency word, yielding low predictability. Conversely, some sentences are highly predictable but implausible, although these types of sentences tend to be exceptions (e.g., idioms, whose functional meaning differs from what a literal composition of each word would suggest). Despite this distinction, plausibility and predictability appear to be overall correlated in language use because language often describes plausible events (that have occurred, will occur, or can be imagined). Indeed, LLMs, by simply learning patterns of word predictability, can acquire some aspects of common‐sense or world knowledge about plausibility (Hu, Sosa, & Ullman, 2025; Kauf, Chersoni, Lenci, Fedorenko, & Ivanova, 2024). On the one hand, such knowledge demonstrates the predictability‐plausibility correlation; on the other hand, LLMs remain sensitive to the distinction between these two constructs, and may process “semantically unlikely” (unpredictable) sentences differently from “semantically implausible” sentences (e.g., those that violate selectional restrictions) (Kauf et al., 2023).

What mechanisms are responsible for the effects of syntactic penetrability that we observed in LLMs? In the human language system, nonencapsulated syntactic processing can be implemented in different ways. First, a system that processes nonsyntactic information can send its output to the syntactic parser and bias its computations; such an architecture seems plausible for the case of visual information penetrating syntactic processing (Tanenhaus et al., 1995), because the visual system and the parser are likely functionally separate systems operating on distinct representations. Second, a shared mechanism can process both syntax and nonsyntactic information in an integrated manner. This latter architecture perhaps describes how syntactic and semantic information are processed, as suggested by neuroimaging research demonstrating that the human “core language network” shows distributed and overlapping sensitivity to syntax and semantics at both the regional level (as measured by functional magnetic resonance imaging) and at a more local resolution (as measured by single electrodes) (Fedorenko et al., 2016, 2020, 2024; Shain et al., 2024). It is also supported by aphasia studies that argue against a modular grammar‐lexicon distinction (Bates & Goodman, 1997). Do LLMs implement penetrable syntactic processing in a manner that mirrors the human mind and brain? Our results do not answer this question, because we characterized penetrability at the representational level, but not at the mechanistic level. Therefore, a future direction of this work is a detailed mapping of how syntactic penetrability to different information sources is implemented in LLMs, for example, with methods such as circuit tracing (Conmy, Mavor‐Parker, Lynch, Heimersheim, & Garriga‐Alonso, 2023; Wang, Variengien, Conmy, Shlegeris, & Steinhardt, 2022).

Beyond the issue of encapsulation versus penetrability, our findings are also consistent with noisy channel processing accounts of human language comprehension. Such accounts acknowledge that signals sent over a communication channel are susceptible to corruption due to noise, and, therefore, the signal perceived by the comprehender is not necessarily identical to the signal intended by the producer (Shannon, 1949). Hence, comprehension involves an inferential process of recovering intended messages from the input (Gibson, Bergen, & Piantadosi, 2013; Levy, Bicknell, Slattery, & Rayner, 2009). Given an implausible sentence such as “*The guide showed the sculpture a visitor*,” a rational comprehender might “mentally correct” the sentence into a more semantically plausible alternative that is only slightly different in surface form, such as “*the guide showed the visitor a sculpture*” or “*the guide showed the sculpture to a visitor*.” Behavioral evidence indicates that humans behave in this way (Cai, Zhao, & Pickering, 2022; Gibson et al., 2013; Poppels & Levy, 2016; Ryskin, Futrell, Kiran, & Gibson, 2018). Our findings suggest that a similar process might take place in LLMs: attention is higher toward a lure in the implausible versus plausible sentences, and we might interpret this pattern as an “inference” that the lure in implausible sentences might be part of the syntactic dependency even though its position is syntactically incorrect; in other words, the attention heads assign some probability to an intended message that is a “corrected” version of the input.

Three issues regarding our study are worth addressing. First, in human language processing, the question of syntactic encapsulation concerns timing: not *whether* semantic information can influence syntactic analysis (it does, because different information sources must interact at some point), but rather *how soon*. Namely, nonencapsulated syntactic mechanisms are those that are penetrable immediately, from the very first moments of processing. However, unlike humans, transformers process all words in parallel rather than incrementally. Does this mean that our results do not really address the critical aspect of encapsulation, that is, timing? We do not think so: the analysis of the unidirectional LLMs (GPT‐2 small and Llama 2) found plausibility effects at the word that constitutes a critical dependency, a site where any words beyond that dependency are unavailable. This is akin to reading times studies with human participants, which have operationalized immediate effects as effects at a critical word—rather than later, at a spillover region—and interpreted these effects as evidence for the early influence of nonsyntactic information (e.g., Trueswell et al., 1994).

A second issue is that our results do not merely reflect general parsing difficulty in an encapsulated syntactic mechanism (instead of the use of semantic information in syntactic computations). One could imagine that “syntax‐specialized” attention heads behaved more randomly when processing implausible versus plausible sentences, distributing attention more evenly across tokens. Given that, in plausible sentences, these heads assign a particularly high weight to the critical dependency, a more even distribution of attention in implausible sentences would result in decreased attention to that dependency, which is the pattern we observed. However, our analysis of lure words indicates that attention weights do not necessarily become more evenly distributed in implausible sentences: in such sentences, attention directed to the lure word increased in comparison to plausible sentences, while attention strength to the unrelated control word did not differ between plausibility conditions (note that this analysis is more specific than an entropy‐based analysis over all attention weights directed from the critical token). Moreover, our implausible sentences were not completely nonsensical sentences from which it would be difficult to extract syntactic or semantic roles; rather, implausibility was limited to a single dependency between two words (or two dependencies in the case of Direct Object and Indirect Object, which used the same stimuli). Indeed, for most dependencies, attention to the syntactically correct (but implausible) word is still higher than attention to the syntactically incorrect (but plausible) lure. These arguments all lend support to our interpretation of the data as reflecting syntactic penetrability to semantic information.

The third issue regards our use of the term “plausibility.” In using this term, we do not commit to attributing “understanding” to LLMs. Whether LLMs have conceptual understanding and “common sense” knowledge is an ongoing debate (e.g., Chang & Bergen, 2024; Mahowald et al., 2024; Pavlick, 2022). In the current study, plausibility is a human‐interpretable feature that distinguishes between our two conditions (i.e., classes of stimuli), and those conditions differ in the patterns of attention they elicit in LLMs; but this does not necessarily mean that LLMs have some explicit representation of plausibility (or of some other semantic variable that correlates with plausibility). Still, our results are consistent with previous claims that plausibility is an organizing dimension of LLM sentence representations (Kauf et al., 2023). LLMs are trained on an objective of word prediction from sequences of words, so whatever factor modulates their attention weights in our experiment must be learnable from co‐occurrence patterns of word forms. Some scholars might claim that any knowledge gained from information about forms lacks semantics (e.g., Bender, Gebru, McMillan‐Major, & Shmitchell, 2021), whereas others would be open to attributing semantic understanding to LLMs (e.g., Merrill, Warstadt, & Linzen, 2022; Piantadosi & Hill, 2022). Our claim here is that a manipulation of information that is usually considered to be outside the domain of syntax, and which influences syntactic processing in humans, also influences syntactic processing in LLMs.

LLMs may be a tool for inferring (or constraining) the algorithms and representations supporting language in the human mind. To use LLMs as models of human language processing in this way, we must first establish whether LLMs and humans align in how they operate on linguistic input. Our study contributes to this question by establishing such an alignment at the “computational level” of analysis (Marr, 1982): syntactic processing in LLMs, like in the human mind, is penetrable to semantic information rather than encapsulated (even if the specific parsing algorithms diverge between brains and machines). Whereas we rely on previous work that localized specific linguistic functions to particular attention heads (e.g., Clark et al., 2019; Vig & Belinkov, 2019), we go beyond such work and characterize one feature of this processing mechanism. In characterizing how dependencies are processed in attention heads, we focus on a central property of the functional architecture—penetrability—thus complementing studies that focus on finer‐grained details and relate the operations of attention heads to an LLM's output (e.g., Wang et al., 2022).

Why might “syntax‐specialized” attention heads in transformer‐based LLMs be penetrable to semantic information, rather than encapsulated? One reason is that encapsulated systems are mostly advantageous in domains whose input is unambiguous, because encapsulation allows a system's expertise to operate without external interference. In contrast, informational domains that are pervasively ambiguous—such as syntax—benefit from penetrability to external information sources that can aid analysis on a first pass and resolve indeterminacies without the need for reprocessing (Trueswell et al., 1994). Given that (1) LLMs are trained to predict words in language, (2) such prediction can be optimized when using syntactic information (e.g., Futrell et al., 2019), and (3) syntactic information is often ambiguous, LLMs might automatically learn to represent and process syntax in a nonencapsulated manner. In this sense, a fundamental computational property of their attention heads shows functional similarities to the human mind.

## CRediT statement

Conceptualization—T.M., I.A.B; Data curation—T.M.; Formal analysis—T.M., Y.Z.; Investigation—T.M., Y.Z.; Methodology—T.M., I.A.B; Project administration—I.A.B; Resources—I.A.B; Software—T.M.; Supervision—I.A.B; Visualization—T.M.; Writing – original draft—T.M., I.A.B; Writing – review & editing—T.M., I.A.B.

## Ethics approval statement

All experiments were performed in compliance with the ethical guidelines of the North General Institutional Review Board at the University of California, Los Angeles.

## Conflict of interest disclosure

We declare no conflicts of interest.

## Data and code availability

Stimuli, data, and code for replicating the results of this experiment are available through the following OSF link. Upon publication, all materials will be publicly accessible via GitHub.

## Supporting information



Supporting Information

## Notes

1 Encapsulation is often confused with the notion of domain‐specificity, but these are two different properties. A system can be domain‐specific—that is, exclusively process and output representations in a particular domain—while still being penetrable to external systems that can influence its operations. Several systems in the human mind, including the language system, appear to behave this way (Tuckute, Kanwisher, & Fedorenko, 2024).

2 One example for syntactic structures being associated with their own meaning, beyond the meaning of the individual words that instantiate those structures, is the difference between the direct‐object (DO) and prepositional object (PO) constructions: “I gave the book to Alice” versus “I gave Alice the book.” Whereas traditional linguistic theories assumed that both surface forms had the same “deep structure”—and thus the same meaning—they in fact differ in meaning. The DO structure has a prototypical meaning of “transfer of possession,” whereas the PO structure has a prototypical meaning of “change in location.” Therefore, we can say “I brought a fork to the table” (change in location), but “I brought the table a fork” is less acceptable (a table cannot be in possession of something). Similarly, the meaning associated with the DO construction explains why we understand a sentence like “He sneezed the napkin off the table” (Goldberg, 1995), which implies caused motion; this meaning cannot come from the words themselves, because the verb “sneezed” is intransitive and thus cannot clarify the roles played by the words that follow it.

3 Whereas attention layers apply linear transformations to their inputs, feedforward layers (also called multilayer perceptron [MLP] layers) apply both linear and nonlinear transformations to their inputs.

4 Bau (2022) investigated how syntactic and semantic corruptions and their combinations influence log‐likelihoods that LLMs assign to tokens, in order to determine the degree to which these domains are “entangled” in LLMs. However, that study did not perform targeted investigations of specific LLM components (e.g., neurons, attention heads, etc.).

5 Measure (2) is identical to Clark, Khandelwal, Levy, and Manning (2019), but the head numbers we identified are different because we use 0‐based indexing, whereas they used 1‐based indexing. Measure (1) differs from Clark et al. (2019): for head→dep, they computed the percentage of instances for which the dependent token *received* more attention weight from the head than *from* any other word in the sentence. We modified this measure to make it analogous to measure (2) (dep→head).

6 As described below, for the Direct Object dependency, we predicted that in implausible compared to plausible sentences, attention from the direct object to the verb would be weaker, and attention from the indirect object (the “lure”) to the verb would be higher. For the Indirect Object dependency, we predicted the reverse pattern. However, because the same attention head appeared specialized for both dependencies in GPT‐2 small, confirming one prediction would disconfirm the other, so we excluded the Indirect Object dependency from analysis.

7 For this analysis, we only tested the single most specialized head for each dependency in the three LLMs.

8 For Nominal Subject, a fragment like “*the artist by the window painted* …” is temporarily ambiguous between an active transitive construction (“…*a landscape*.”) and the beginning of a reduced relative clause (“…*by the student was very still*.”). For this dependency in GPT‐2 small and Llama2, our stimuli reflect more closely the kind of stimuli used with human participants in prior work, which are often syntactically ambiguous.
