# Supplementary material for: Evidence Against Syntactic Encapsulation in Large Language Models
Source: Cogn Sci. 2026 Mar 10;50(3):e70187. doi: 10.1111/cogs.70187 (PMC12973484; doi:10.1111/cogs.70187)
Supplement: Supplementary file 1 — Supporting Information [file COGS-50-e70187-s001.pdf]

Appendix A

Human plausibility experiment: plausibility validation and attention strength prediction

In a supplementary experiment, we tested whether human plausibility ratings of individual sentences predicted attention strength from GPT-2 small for syntactic dependencies within the same sentences.

Participants

We recruited 84 participants (all undergraduate students) via the university's participant recruitment system. All were fluent in English and provided informed consent before participation. The study received approval from the relevant institutional review board (IRB) to ensure compliance with ethical research standards.

Stimuli

To obtain a larger range of plausibility values compared to the experiments reported in the manuscript, we added two sentence conditions designed to have "intermediate" plausibility. We label the resulting 4 conditions "Very Implausible", "Implausible", "Plausible", and "Very Plausible". Each plausibility condition comprised 40 sentences. The Very Implausible and Very Plausible conditions were based on a subset of the stimuli from the original materials. Table A1 shows example stimuli.

Table A1

Example stimuli: four-condition minimal sets for each dependency (for GPT-2)

Dependency	Very Plausible	Plausible	Implausible	Very Implausible
Direct Object	The guide **showed** the visitor a **<u>sculpture</u>**.	The guide **showed** the visitor a **<u>bell</u>**.	The guide **showed** the visitor a **<u>meal</u>**.	The guide **showed** the sculpture a **<u>visitor</u>**.
Adverb Modifier	The species that is **threatened <u>terribly</u>** is rarely seen.	The species that is **taught <u>terribly</u>** is rarely seen.	The species that is **cooked <u>terribly</u>** is rarely seen.	The species that is **seen <u>terribly</u>** is rarely threatened.
Nominal Subject	It was a landscape that the **artist** by the window **<u>painted</u>**.	It was a landscape that the **artist** by the window **<u>imagined</u>**.	It was a landscape that the **artist** by the window **<u>destroyed</u>**.	It was a landscape that the **window** by the artist **<u>painted</u>**.
Passive Nominal Subject	The **house** in the novel was **<u>sold</u>**.	The **house** in the novel was **<u>scratched</u>**.	The **house** in the novel was **<u>pushed</u>**.	The **house** in the novel was **<u>read</u>**.
Object of Preposition	The child walked **across** the city's recently made **<u>streets</u>**.	The child walked **across** the city's recently made **<u>fountain</u>**.	The child walked **across** the city's recently made **<u>pillow</u>**.	The child walked **across** the city's recently made **<u>absence</u>**.

Note: Words in bold constitute the critical dependency. Attention is directed from the underlined word to the other bolded word. For the Nominal Subject dependency, the sentence structure was modified so that the syntactic head appeared as the final word, ensuring comparable context for GPT-2 small and human participants. For the Passive Nominal Subject dependency, text following the syntactic head from the original stimuli was removed so that both GPT-2 small and human participants received comparable context.

Procedure and Results

Each participant rated 100 sentences, evenly distributed across plausibility levels and syntactic dependencies, on a 5-point Likert scale ranging from "Very Implausible" (1) to "Very Plausible" (5). Sentences were presented in randomized order. To account for participant-specific strategies of using the plausibility scale, ratings were *z*-scored within each participant.

For nearly all dependencies, plausibility ratings differed significantly between adjacent pairs of conditions, in the expected direction, as indicated by Bonferroni-corrected, one-tailed, paired-samples *t*-tests (results and significance displayed in Figure A1). The only exceptions were the plausible vs. very plausible comparison for the Adverb Modifier ($t_{(39)} = -4.84$, $p = 1$) and the Passive Nominal Subject ($t_{(39)} = 2.16$, $p = 0.019$) ($\alpha = 0.05/5$).

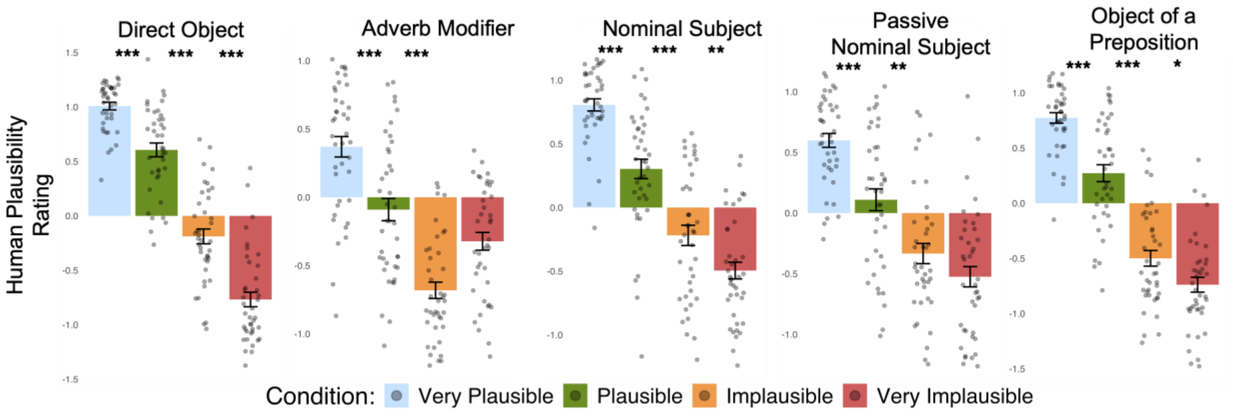

Figure A1: Mean human plausibility ratings for the four plausibility conditions tested. Dots correspond to the mean *z*-scores of the plausibility ratings for each sentence, with bars showing averages, and error bars representing standard errors. The significance of Bonferroni-corrected p-values (i.e., after multiplying the reported p-values by the number of comparisons) is reflected above the plots: * for $p<0.05$; ** for $p< 0.01$; and *** for $p<0.001$.

We tested whether sentence-level human plausibility ratings predicted attention strength from GPT-2 small using a linear, mixed effect regression model. The model included fixed effects of both "plausibility condition" (i.e., Very Plausible, Plausible, Implausible, Very Implausible) and "human plausibility rating", as well as a random intercept and a slope of human ratings by item (i.e., a quadruplet of sentences across the 4 conditions). The significance of each fixed effect

was tested via a likelihood ratio test that compared this full model to a nested model that did not include the relevant fixed effect but was otherwise identical. Statistics for the individual models are displayed in Table A2.

For all dependencies, human plausibility ratings did not explain variance in attention strength over and above the condition labels (Direct Object: $\chi^2(1) = 0.0022$, $p = 0.96$; Adverb Modifier: $\chi^2(1) = 0.0037$, $p = 0.95$; Nominal Subject: $\chi^2(1) = 0.075$, $p = 0.78$; Passive Nominal Subject: $\chi^2(1) = 2.22$, $p = 0.14$; Object of a Preposition: $\chi^2(1) = 0.013$, $p = 0.91$). However, condition labels did explain variance in attention strength over and above human ratings for 3 dependencies (Direct Object: $\chi^2(1) = 7.52$, $p = 0.0061$; Adverb Modifier: $\chi^2(1) = 0.088$ $p = 0.77$; Nominal Subject: $\chi^2(1) = 8.10$, $p = 0.0044$; Passive Nominal Subject: $\chi^2(1) = 4.85$, $p = 0.028$), but not for the Nominal Subject ($\chi^2(1) = 0.075$, $p = 0.78$) or Object of a Preposition dependencies ($\chi^2(1) = 1.65$, $p = 0.20$). Note that, by design, human ratings were strongly correlated with condition labels (Figure A1); indeed, in the 3 dependencies where attention strength was significantly predicted by condition labels in the model without human ratings (Direct Object, Nominal Subject, Passive Nominal Subject), it was also significantly predicted by human ratings in the model without condition labels. Overall, these results suggest that whereas attention strength is sensitive to semantic plausibility, it does not co-vary with human judgments at the individual sentence level. One reason might be that, whereas our dependent measure in LLMs was attention strength to a specific syntactic dependency, human plausibility judgments evaluate a sentence as a whole, not just a particular dependency.

Table A2: Statistics for linear mixed-effects models containing fixed effects for both “human plausibility rating” and “plausibility condition” (full model), or only one of the two effects (reduced models).

**Dependency**	**Model and fixed effect**	***b***	***SE***	**df**	***t*-value**	***p*-value**
Direct Object	Reduced model: human plausibility rating	0.068	0.014	34.52	4.58	2.6×10^{-5}
Direct Object	Reduced model: Plausibility condition	-0.051	0.0089	64.98	-5.77	2.37×10^{-7}
Adverbial Modifier	Reduced model: human plausibility rating	0.0063	0.018	32.47	0.34	0.73
Adverbial Modifier	Reduced model: Plausibility condition	-0.0039	0.0085	98.40	-0.46	0.65
Nominal Subject	Reduced model: human plausibility rating	0.055	0.018	91.16	3.044	0.0031
Nominal Subject	Reduced model: Plausibility condition	-0.041	0.0096	108.59	-4.28	4.09×10^{-5}
Passive Nominal Subject	Reduced model: human plausibility rating	0.057	0.016	33.66	3.53	0.0012
Passive Nominal Subject	Reduced model: Plausibility condition	-0.027	0.007	112.43	-3.86	0.00019
Object of a Preposition	Reduced model: human plausibility rating	0.0092	0.0044	77.42	2.062	0.043
Object of a Preposition	Reduced model: Plausibility condition	-0.0067	0.003	98.29	-2.42	0.017

Appendix B

Supplementary Experiment: Contrasting Plausibility and Predictability

During training, LLMs are likely to see words occupying dependencies in semantically plausible configurations (like the critical dependencies in our plausible condition) but might only infrequently be exposed to words occupying semantically implausible dependencies (like in our implausible condition). For example, LLMs have more experience with a Nominal Subject dependency between "artist" and "painted" and less experience with that dependency between "window" and "painted"; as a result, encountering "painted" as the verb of the subject "artist" in our plausible condition would be more predictable than encountering it as the verb of the subject "window" in our implausible condition. This difference in predictability, resulting from a difference in frequency of exposure, could drive differences in attention strength between our two conditions. Therefore, we evaluated the extent to which any effects of plausibility in our analyses could be accounted for by effects of predictability.

Stimuli

To disentangle the effects of plausibility vs. predictability, we added new stimuli to obtain a wider range of predictability values for plausible sentences. To this end, we created a third experimental condition, in which the original sentences from the plausible condition were edited to have the critical word in the dependency be highly surprising but still plausible (e.g., "*the artist by the window* ***imagined*** *a landscape*" vs. the original "*painted*"). We directly selected critical words that were unpredictable in the context of the sentence, rather than simply selecting, e.g., low frequency words, by computing the surprisal of those words ($-\log(p(word|context))$; Hale, 2001; Levy, 2008) from the last layer of GPT-2 small.

Figure B1 shows the surprisal values of the critical words in these plausible-surprising sentences, as well as the surprisal values of the original two conditions (plausible, implausible). The average surprisal was significantly greater in the plausible-surprising condition than in the original plausible condition for four out of five dependencies, as indicated by permutation tests (Direct Object: mean difference = 2.97 bits, $p = 0$; Nominal Subject: mean difference = 3.01 bits, $p = 0$; Passive Nominal Subject: mean difference = 1.72 bits, $p = 8 \times 10^{-4}$; Object of a Preposition: mean difference = 2.82 bits, $p = 0$). For the Adverb Modifier dependency, average surprisal in the plausible-surprising condition was not significantly greater following Bonferroni correction ($\alpha = 0.05/5$; mean difference = 0.50 bits, $p = 0.028$). Plausible-surprising sentences were not always as

surprising as the implausible sentences (Passive Nominal Subject: mean difference = -2.30 bits, p = 0; Object of a Preposition: mean difference = -4.66 bits, p = 0). For the remaining dependencies, there were no significant differences between plausible surprising and implausible sentences, following Bonferroni correction (α = 0.05/5; Direct Object: mean difference = 0.49, p = 0.33; Adverb Modifier: mean difference = -0.52, p = 0.03; Nominal Subject: mean difference = -0.31, p = 0.41). Finally, average surprisal was significantly greater in the implausible condition than in the original plausible condition for four dependencies, following Bonferroni correction (α = 0.05/5; Direct Object: mean difference = 2.48 bits, p = 0; Adverb Modifier: mean difference = 1.02 bits, p = 0.0012; Nominal Subject: mean difference = 3.32 bits, p = 0; Passive Nominal Subject: mean difference = 4.02 bits, p = 0; Object of a Preposition: mean difference = 7.48 bits, p = 0). We emphasize that plausibility was judged by the authors based on intuition, rather than based on empirical judgments from participants.

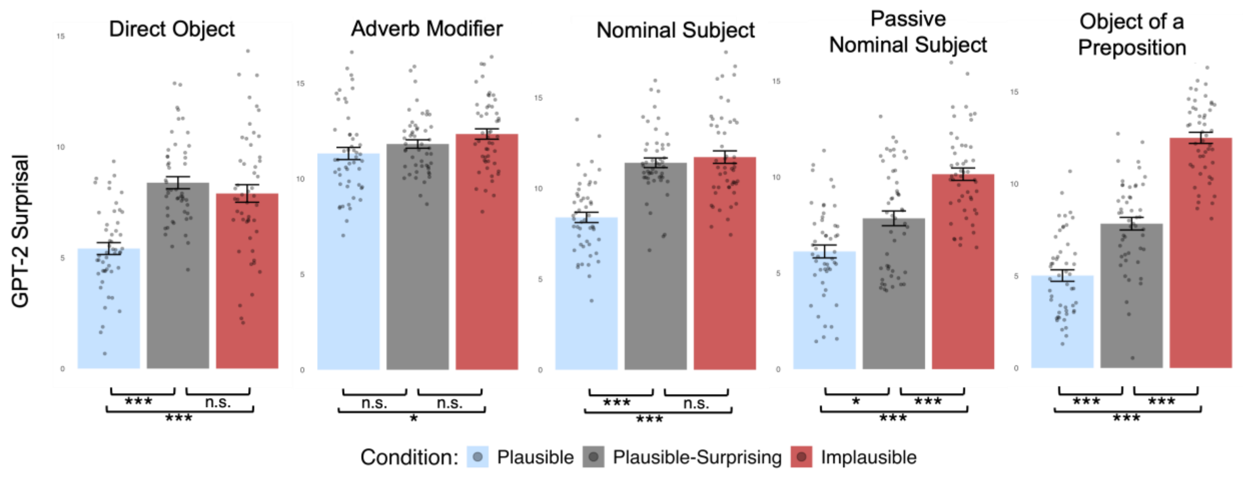

Figure B1. Average surprisal values (from GPT-2 small) for the critical word in the plausible (blue), plausible-surprising (gray), and implausible (red) conditions. Each panel displays data for a different syntactic dependency. Dots correspond to individual sentences, with bars showing averages, and error bars—standard errors. The significance of Bonferroni-corrected p-values (i.e., after multiplying the reported p-values by the number of comparisons) is reflected beneath the plots: * for $p<0.05$; ** for $p< 0.01$; and *** for $p<0.001$.

Analysis

We used the same approach as in the main analysis to extract, for each sentence in each condition, attention weights from the "syntax-specialized" attention heads. We tested whether attention strength varied across conditions by fitting a linear, mixed-effects regression model to logit-

transformed attention weights. The model included plausibility as a fixed effect with two levels (one coding the implausible condition, and one coding both the plausible and plausible-surprising conditions), surprisal as a continuous covariate, and an intercept by sentence triplet. As above, we corrected for multiple comparisons across dependencies, separately for each statistical model.

Results

Attention strength for the critical dependency in GPT-2 small was significantly higher in the plausible conditions than in the implausible condition, while controlling for surprisal, for 3 out of 5 dependencies: Direct Object (b = 0.45, SE = 0.10, $t_{(98.52)}$=4.43, $p<10^{-4}$), Nominal Subject (b = 0.63, SE = 0.13, $t_{(107.86)}$=4.65, $p<10^{-4}$), and Passive Nominal Subject (b = 0.30, SE = 0.11, $t_{(104.17)}$ = 2.77, p=0.00329). This pattern did not hold for Adverb Modifier (b = -0.25, SE = 0.14, $t_{(103.65)}$ = -1.82, p=0.036) and Object of a Preposition (b = 0.13, SE = 0.085, $t_{(113.15)}$ = 1.58, p = 0.059). The results are shown in **Figure B2**.

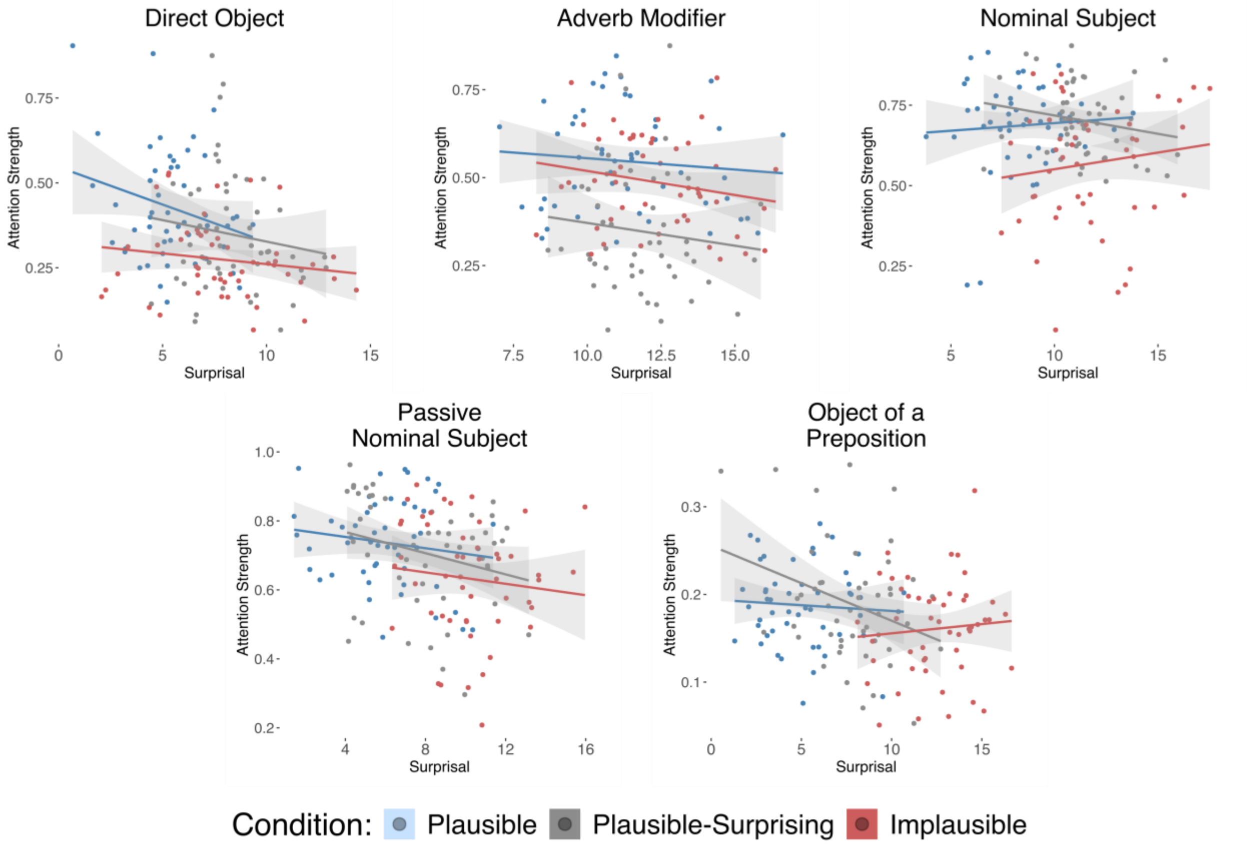

Figure B2. Attention strength between words in a critical dependency (extracted from "syntax-specialized" attention heads in GPT-2) by GPT-2-based surprisal. Each dot corresponds to an individual sentence from one of three conditions: plausible (blue), plausible-surprising (gray), and implausible (red) conditions. Lines of best fit are

displayed for each condition within a dependency, and the gray border represents the confidence interval for each regression line.

Appendix C

Results for a second Object of a Preposition construction for BERT

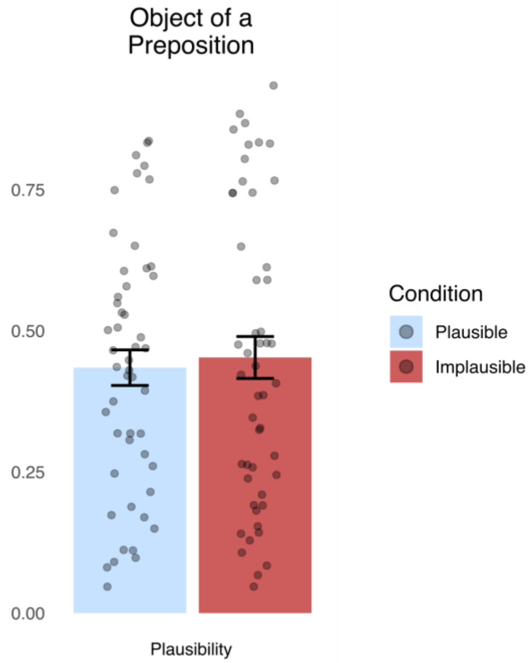

Figure C1. We tested BERT on an additional Object of a Preposition sentence structure, which was used for GPT-2 and Llama 2; for this structure, attention strength was not significantly greater in the plausible condition (b = -0.087, SE = 0.19, $t_{(47.81)}$ = -0.45, p=0.33).

Appendix D

Syntax-Specialized Attention Heads in BERT, GPT-2 Small, and Llama2

Table D1: Attention heads specialized for different dependencies. We include here attention heads whose accuracy score for the dependency of interest was at least 10% higher than the fixed offset baseline accuracy. For dependencies for which more than five attention heads exceed this threshold, we tested the 5 heads with the highest accuracy scores.

BERT

Relation	Head	Direction	Specialization score	Baseline score (distance)
Direct Object	7-9	dep→head	86.47	39.78 (-2)
Direct Object	6-9	dep→head	80.23	39.78 (-2)
Direct Object	3-11	dep→head	67.50	39.78 (-2)
Direct Object	4-5	head→dep	62.55	39.78 (2)
Direct Object	7-10	head→dep	62.12	39.78 (2)
Adverb Modifier	7-3	head→dep	51.97	41.35 (-1)
Nominal Subject	7-1	dep→head	60.89	46.46 (1)
Nominal Subject	7-11	head→dep	58.41	46.46 (-1)
Passive Nominal Subject	4-10	head→dep	62.45	43.87 (-2)
Passive Nominal Subject	8-10	head→dep	56.92	43.87 (-2)
Passive Nominal Subject	6-5	dep→head	58.50	43.87 (2)
Object of Preposition	7-10	head→dep	81.95	34.67 (2)
Object of Preposition	4-5	head→dep	74.73	34.67 (2)
Object of Preposition	8-5	head→dep	66.10	34.67 (2)
Object of Preposition	5-0	head→dep	65.01	34.67 (2)
Object of Preposition	6-9	dep→head	69.75	34.67 (-2)
Indirect Object	6-9	dep→head	77.07	46.34 (-1)
Indirect Object	5-10	dep→head	71.22	46.34 (-1)
Indirect Object	3-11	dep→head	63.90	46.34 (-1)
Indirect Object	3-9	head→dep	75.12	46.34 (1)
Indirect Object	5-9	head→dep	74.63	46.34 (1)

GPT-2 small

Relation	Head	Direction	Specialization score	Baseline score (distance)
Direct Object	2-8	dep→head	81.63	40.63 (-2)
Direct Object	4-0	dep→head	76.25	40.63 (-2)
Direct Object	3-8	dep→head	53.81	40.63 (-2)
Adverb Modifier	2-8	dep→head	65.24	52.14 (-1)
Nominal Subject	4-3	head→dep	66.87	48.66 (-1)
Nominal Subject	2-9	head→dep	59.84	48.66 (-1)
Passive Nominal Subject*	4-3	head→dep	73.31	44.20 (-2)
Object of Preposition	2-0	dep→head	76.55	34.62 (-2)
Object of Preposition	3-8	dep→head	76.15	34.62 (-2)
Object of Preposition	2-8	dep→head	72.35	34.62 (-2)
Object of Preposition	4-0	dep→head	71.25	34.62 (-2)
Object of Preposition	2-5	dep→head	50.30	34.62 (-2)

Llama 2

Relation	Head	Direction	Specialization score	Baseline score (distance)
Direct Object	4-11	dep→head	74.63	40.63 (-2)
Direct Object	3-11	dep→head	71.19	40.63 (-2)
Direct Object	10-17	dep→head	67.25	40.63 (-2)
Direct Object	22-21	dep→head	65.56	40.63 (-2)
Direct Object	24-10	dep→head	65.00	40.63 (-2)
Nominal Subject	3-22	head→dep	65.06	48.66 (-1)
Nominal Subject	4-30	head→dep	64.30	48.66 (-1)
Nominal Subject	17-15	head→dep	61.35	48.66 (-1)
Nominal Subject	8-1	head→dep	60.03	48.66 (-1)
Nominal Subject	5-18	head→dep	58.86	48.66 (-1)
Passive Nominal Subject*	4-30	head→dep	70.50	44.22 (-2)
Passive Nominal Subject*	5-18	head→dep	67.33	44.22 (-2)
Passive Nominal Subject*	4-28	head→dep	64.54	44.22 (-2)
Passive Nominal Subject*	3-22	head→dep	60.16	44.22 (-2)
Passive Nominal Subject*	9-2	head→dep	59.76	44.22 (-2)
Object of Preposition*	10-17	dep→head	67.02	34.60 (-2)
Object of Preposition*	3-11	dep→head	63.61	34.60 (-2)
Object of Preposition*	6-3	dep→head	52.89	34.60 (-2)
Object of Preposition*	31-13	dep→head	52.79	34.60 (-2)
Object of Preposition*	12-29	dep→head	51.74	34.60 (-2)

Appendix E

The Distribution of Syntax-Specialized Attention Heads in BERT, GPT-2 Small, and Llama2

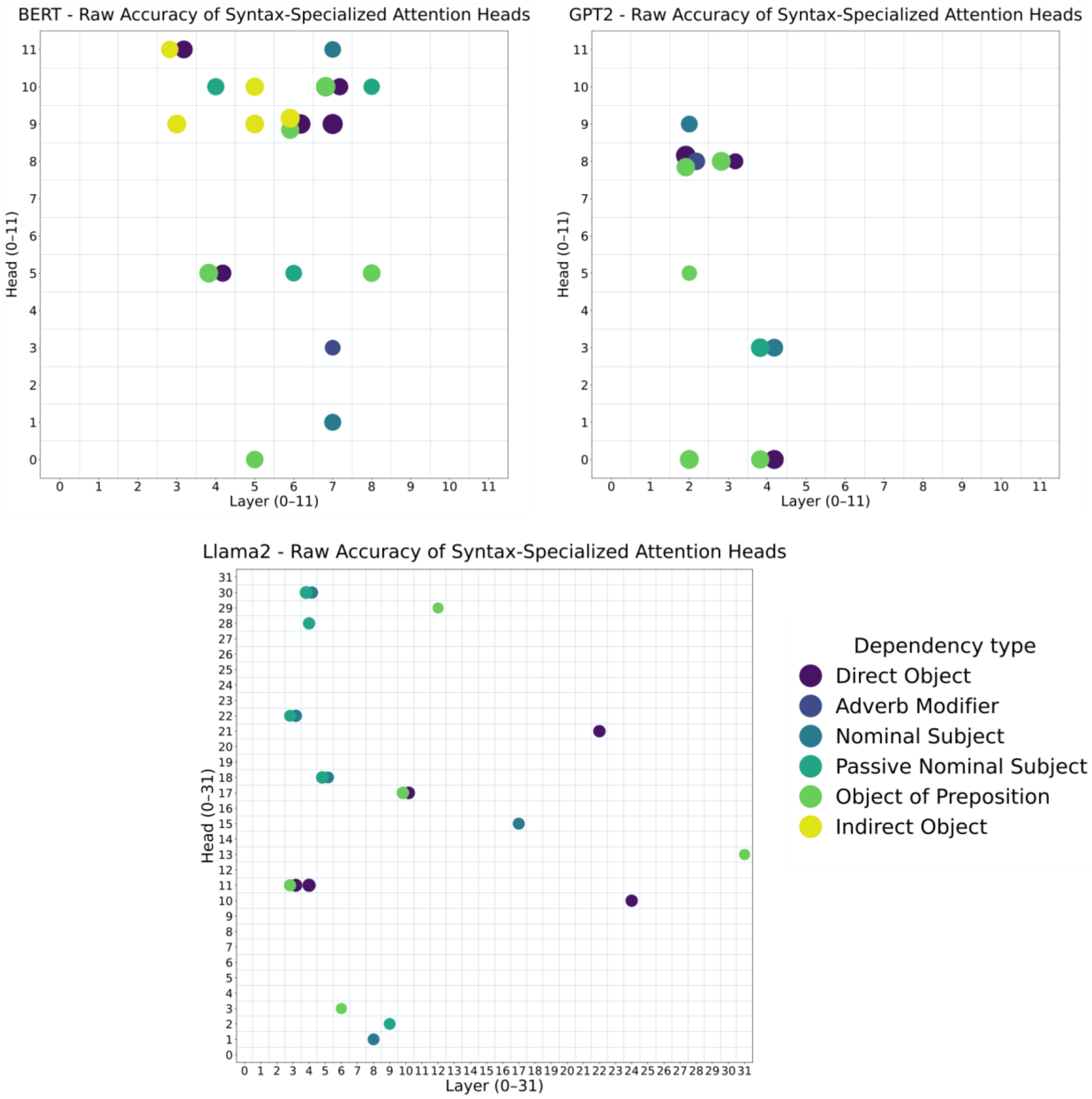

Figure E1. Syntax-specialized attention heads shown by head and layer, where a head is syntax-specialized if its accuracy score was at least 10% greater than the fixed offset baseline score. If more than 5 attention heads met this threshold for syntax specialization for a given model-dependency pair, we only include the top 5 heads with the highest specialization scores. The color of the dot corresponds to the dependency for which a head is specialized, and the size of the dot corresponds to its absolute accuracy score. While accuracy scores can be compared based on relative size *within* each plot (model), to compare accuracies across models refer to Table D1. If a head was specialized for multiple syntactic dependencies, we display this using overlapping circles within the relevant cell. Interestingly, across all three models, we see that the most frequent pair of dependencies that is encoded within the same attention head is the Direct Object and the Object of a Preposition dependencies. Additionally, in BERT there are two cases in which the same attention head is specialized for the direct and indirect object dependencies, and one of these heads (head 6-9) is

specialized for the direct, indirect object, and object of a preposition dependencies. Furthermore, in GPT-2 and Llama2, we found that various heads co-represent the nominal subject and passive nominal subject dependencies. This suggests that some attention heads may encode the nominal and passive nominal subjects as the same type of syntactic dependency that can appear in different syntactic contexts (i.e., constructions).

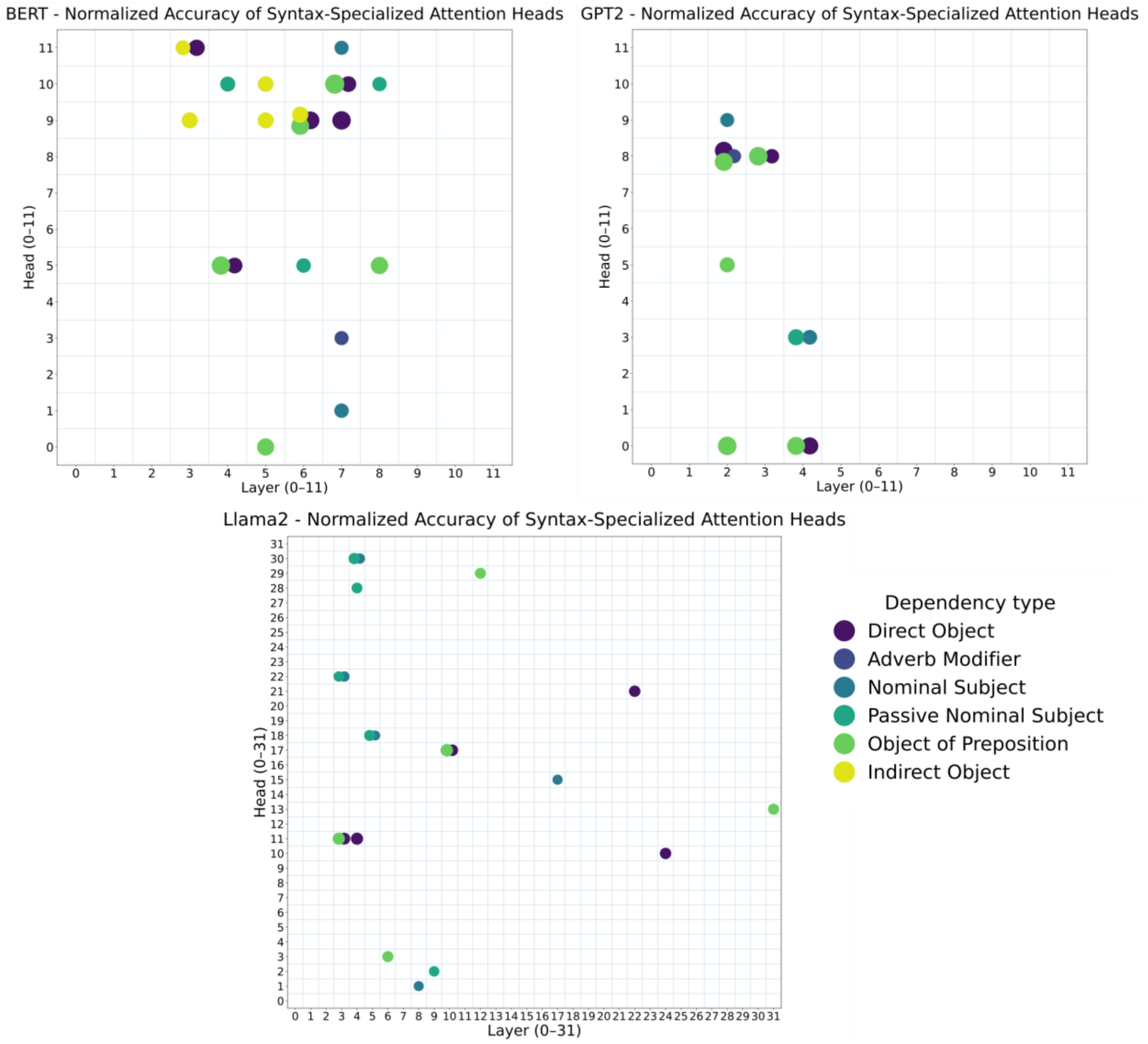

Figure E2. Syntax-specialized attention heads shown by head and layer, where a head is syntax-specialized if its accuracy score was at least 10% greater than the fixed offset baseline score. If more than 5 attention heads met this threshold for syntax specialization for a given model-dependency pair, we only include the top 5 heads with the highest specialization scores. The color of the dot corresponds to the dependency for which a head is specialized, and the size of the dot corresponds to its normalized accuracy score. Normalized accuracy scores were computed by dividing the absolute accuracy score for a given head by the fixed baseline accuracy score for the relevant dependency. While accuracy scores can be compared based on relative size *within* each plot (model), to compare accuracies across models refer to Table D1. If a head was specialized for multiple syntactic dependencies, we display this using overlapping circles within the relevant cell.

Appendix F

Results for all Syntax-Specialized Attention Heads for Critical and Lure Analyses

Statistics for linear mixed-effects models containing fixed effects for both Plausibility and log word frequency (simple model), and Plausibility, log word frequency, PMI, and cosine similarity (full model). Benjamini-Hochberg-corrected *p*-values are displayed for both model types. Significant results in the predicted direction are in bold.

Table F1: BERT, main analysis—Full Model

Relation	Head	Average Attention (Plausible)	Average Attention (Implausible)	*b*	*SE*	*df*	*t*	*p*
**dobj**	**7_9**	**0.15**	**0.11**	**0.47**	**0.15**	**53.61**	**3.12**	**0.003**
dobj	6_9	0.07	0.09	-0.25	0.15	50.57	-1.65	0.14
**dobj**	**3_11**	**0.16**	**0.14**	**0.28**	**0.13**	**53.24**	**2.21**	**0.02**
**dobj**	**4_5**	**0.14**	**0.04**	**1.18**	**0.15**	**55**	**7.72**	**< .0001**
**dobj**	**7_10**	**0.1**	**0.04**	**0.86**	**0.21**	**53.58**	**4.11**	**< .001**
advmod	7_3	0.08	0.07	0.17	0.15	52.51	1.15	0.16
**nsubj**	**7_1**	**0.19**	**0.14**	**0.43**	**0.09**	**49.08**	**4.92**	**< .0001**
**nsubj**	**7_11**	**0.05**	**0.04**	**0.28**	**0.13**	**47.57**	**2.22**	**0.02**
**nsubjpass**	**4_10**	**0.33**	**0.25**	**0.44**	**0.17**	**72.03**	**2.65**	**0.009**
nsubjpass	8_10	0.17	0.19	-0.1	0.26	95	-0.38	0.71
nsubjpass	6_5	0.06	0.06	-0.14	0.22	55.29	-0.62	0.56
**pobj**	**7_10**	**0.52**	**0.19**	**2.12**	**0.36**	**57.75**	**5.83**	**< .0001**
**pobj**	**4_5**	**0.01**	**0.003**	**1.09**	**0.24**	**58.61**	**4.54**	**< .0001**
**pobj**	**8_5**	**0.31**	**0.2**	**0.7**	**0.25**	**56.69**	**2.8**	**0.007**
pobj	5_0	0.002	0.002	0.12	0.15	60.36	0.83	0.23
pobj	6_9	0.005	0.003	0.16	0.18	60.41	0.91	0.21
iobj	6_9	0.54	0.57	-0.26	0.12	52.72	-2.18	0.05
**iobj**	**5_10**	**0.5**	**0.37**	**0.58**	**0.12**	**51.97**	**4.85**	**< .0001**
**iobj**	**3_11**	**0.48**	**0.38**	**0.25**	**0.1**	**54.19**	**2.44**	**0.02**
**iobj**	**3_9**	**0.63**	**0.44**	**1**	**0.17**	**53.87**	**5.98**	**< .0001**
**iobj**	**5_9**	**0.78**	**0.47**	**1.66**	**0.19**	**54.69**	**8.61**	**< .0001**

Table F2: BERT, main analysis —Simple Model

Relation	Head	Average Attention (Plausible)	Average Attention (Implausible)	*b*	*SE*	*df*	*t*	*p*
**dobj**	**7_9**	**0.15**	**0.11**	**0.54**	**0.15**	**49.6**	**3.64**	**< .001**
dobj	6_9	0.07	0.09	-0.18	0.15	49.27	-1.19	0.28
dobj	3_11	0.16	0.14	0.15	0.13	50.35	1.13	0.17
**dobj**	**4_5**	**0.14**	**0.04**	**1.39**	**0.16**	**50.25**	**8.44**	**< .0001**
**dobj**	**7_10**	**0.1**	**0.04**	**0.91**	**0.2**	**97**	**4.52**	**< .0001**
**advmod**	**7_3**	**0.08**	**0.07**	**0.3**	**0.13**	**48.44**	**2.3**	**0.02**
**nsubj**	**7_1**	**0.19**	**0.14**	**0.41**	**0.09**	**48.37**	**4.73**	**< .0001**
**nsubj**	**7_11**	**0.05**	**0.04**	**0.25**	**0.13**	**48.89**	**1.95**	**0.04**
**nsubjpass**	**4_10**	**0.33**	**0.25**	**0.4**	**0.1**	**48.5**	**3.91**	**< .001**
nsubjpass	8_10	0.17	0.19	-0.17	0.17	97	-1.02	0.33
nsubjpass	6_5	0.06	0.06	-0.13	0.12	47.7	-1.06	0.33
**pobj**	**7_10**	**0.52**	**0.19**	**2.26**	**0.26**	**48.44**	**8.54**	**< .0001**
**pobj**	**4_5**	**0.01**	**0.003**	**1.41**	**0.18**	**48.79**	**7.91**	**< .0001**
**pobj**	**8_5**	**0.31**	**0.2**	**0.97**	**0.18**	**48.78**	**5.26**	**< .0001**
pobj	5_0	0.002	0.002	0.01	0.11	48.94	0.13	0.45
**pobj**	**6_9**	**0.005**	**0.003**	**0.37**	**0.14**	**49.03**	**2.71**	**0.007**
iobj	6_9	0.54	0.57	-0.16	0.11	49.44	-1.37	0.22
**iobj**	**5_10**	**0.5**	**0.37**	**0.52**	**0.12**	**49.66**	**4.47**	**< .0001**
**iobj**	**3_11**	**0.48**	**0.38**	**0.41**	**0.11**	**97**	**3.66**	**< .001**
**iobj**	**3_9**	**0.63**	**0.44**	**0.83**	**0.17**	**49.57**	**4.99**	**< .0001**
**iobj**	**5_9**	**0.78**	**0.47**	**1.57**	**0.18**	**50.28**	**8.78**	**< .0001**

Table F3: GPT-2 Small, main analysis —Full Model

Relation	Head	Average Attention (Plausible)	Average Attention (Implausible)	*b*	*SE*	*df*	*t*	*p*
**dobj**	**2_8**	**0.43**	**0.27**	**0.7**	**0.13**	**53.62**	**5.42**	**< .0001**
**dobj**	**4_0**	**0.52**	**0.28**	**1.27**	**0.18**	**48.96**	**7.13**	**< .0001**
dobj	3_8	0.21	0.24	-0.02	0.16	52.94	-0.13	0.90
advmod	2_8	0.55	0.49	0.19	0.11	53.19	1.69	0.07
**nsubj**	**4_3**	**0.69**	**0.57**	**0.57**	**0.15**	**48.76**	**3.81**	**< .001**
**nsubj**	**2_9**	**0.16**	**0.14**	**0.18**	**0.09**	**95**	**2.04**	**0.04**
**nsubjpass**	**4_3**	**0.74**	**0.63**	**0.59**	**0.21**	**62.47**	**2.86**	**0.007**
pobj	2_0	0.19	0.16	0.12	0.08	60.54	1.65	0.07
pobj	3_8	0.19	0.16	0.24	0.14	58.53	1.72	0.07
pobj	2_8	0.15	0.12	0.09	0.13	61.08	0.69	0.29
**pobj**	**4_0**	**0.48**	**0.33**	**0.64**	**0.2**	**60.61**	**3.22**	**0.003**
pobj	2_5	0.04	0.05	-0.04	0.13	59.96	-0.28	0.85

Table F4: GPT-2 Small, main analysis —Simple Model

Relation	Head	Average Attention (Plausible)	Average Attention (Implausible)	*b*	*SE*	*df*	*t*	*p*
**dobj**	**2_8**	**0.43**	**0.27**	**0.8**	**0.13**	**49.86**	**6.33**	**< .0001**
**dobj**	**4_0**	**0.52**	**0.28**	**1.29**	**0.17**	**48.63**	**7.72**	**< .0001**
dobj	3_8	0.21	0.24	-0.24	0.17	97	-1.42	0.17
**advmod**	**2_8**	**0.55**	**0.49**	**0.26**	**0.1**	**48.02**	**2.49**	**0.01**
**nsubj**	**4_3**	**0.69**	**0.57**	**0.55**	**0.15**	**48.56**	**3.74**	**< .001**
**nsubj**	**2_9**	**0.16**	**0.14**	**0.18**	**0.09**	**97**	**2.03**	**0.03**
**nsubjpass**	**4_3**	**0.74**	**0.63**	**0.54**	**0.12**	**48.18**	**4.62**	**< .0001**
**pobj**	**2_0**	**0.19**	**0.16**	**0.23**	**0.06**	**46.49**	**3.73**	**< .001**
**pobj**	**3_8**	**0.19**	**0.16**	**0.27**	**0.12**	**48.12**	**2.3**	**0.02**
**pobj**	**2_8**	**0.15**	**0.12**	**0.23**	**0.11**	**47.18**	**2.12**	**0.03**
**pobj**	**4_0**	**0.48**	**0.33**	**0.72**	**0.16**	**48.24**	**4.54**	**< .0001**
pobj	2_5	0.04	0.05	-0.1	0.1	48.42	-0.97	0.34

Table F5: Llama2, main analysis —Full Model

Relation	Head	Average Attention (Plausible)	Average Attention (Implausible)	*b*	*SE*	*df*	*t*	*p*
**dobj**	**4_11**	**0.29**	**0.1**	**1.44**	**0.19**	**95**	**7.47**	**< .0001**
**dobj**	**3_11**	**0.1**	**0.07**	**0.29**	**0.1**	**61.97**	**2.76**	**0.006**
**dobj**	**10_17**	**0.04**	**0.03**	**0.3**	**0.16**	**60.06**	**1.87**	**0.04**
dobj	22_21	0.05	0.05	0.04	0.16	61.93	0.24	0.43
dobj	24_10	0.18	0.16	-0.07	0.2	58.31	-0.36	0.72
**nsubjpass**	**4_30**	**0.48**	**0.24**	**1.01**	**0.21**	**66.39**	**4.87**	**< .0001**
nsubjpass	5_18	0.22	0.28	-0.18	0.13	62.45	-1.35	0.20
**nsubjpass**	**4_28**	**0.18**	**0.09**	**0.48**	**0.19**	**69**	**2.52**	**0.009**
nsubjpass	3_22	0.03	0.03	-0.17	0.08	56.12	-2.14	0.04
**nsubjpass**	**9_2**	**0.24**	**0.17**	**0.76**	**0.29**	**71.55**	**2.63**	**0.008**
**nsubj**	**3_22**	**0.11**	**0.06**	**0.67**	**0.06**	**48.68**	**10.78**	**< .0001**
**nsubj**	**4_30**	**0.42**	**0.23**	**1.0**	**0.17**	**95**	**6.02**	**< .0001**
nsubj	17_15	0.22	0.33	-0.71	0.16	48.73	-4.35	< .001
**nsubj**	**8_1**	**0.25**	**0.09**	**1.63**	**0.21**	**95**	**7.76**	**< .0001**
**nsubj**	**5_18**	**0.17**	**0.12**	**0.58**	**0.19**	**95**	**3.05**	**0.002**
**pobj**	**10_17**	**0.31**	**0.23**	**0.57**	**0.15**	**54.89**	**3.74**	**< .001**
**pobj**	**3_11**	**0.13**	**0.07**	**0.6**	**0.09**	**60.45**	**6.68**	**< .0001**
**pobj**	**6_3**	**0.2**	**0.08**	**1.07**	**0.16**	**57.56**	**6.88**	**< .0001**
**pobj**	**31_13**	**0.11**	**0.04**	**1.18**	**0.15**	**57.18**	**7.86**	**< .0001**
**pobj**	**12_29**	**0.18**	**0.04**	**1.77**	**0.19**	**60.88**	**9.1**	**< .0001**

Table F6: Llama2, main analysis —Simple Model

Relation	Head	Average Attention (Plausible)	Average Attention (Implausible)	*b*	*SE*	*df*	*t*	*p*
**dobj**	**4_11**	**0.29**	**0.1**	**1.41**	**0.17**	**97**	**8.24**	**< .0001**
**dobj**	**3_11**	**0.1**	**0.07**	**0.34**	**0.09**	**48.48**	**3.64**	**< .001**
**dobj**	**10_17**	**0.04**	**0.03**	**0.27**	**0.14**	**46.81**	**1.92**	**0.04**
dobj	22_21	0.05	0.05	-0.01	0.14	48.46	-0.04	0.97
dobj	24_10	0.18	0.16	0.13	0.17	48.59	0.76	0.25
**nsubjpass**	**4_30**	**0.48**	**0.24**	**1.23**	**0.15**	**50.12**	**8.39**	**< .0001**
nsubjpass	5_18	0.22	0.28	-0.35	0.09	50.27	-3.87	< .001
**nsubjpass**	**4_28**	**0.18**	**0.09**	**0.9**	**0.14**	**50.64**	**6.51**	**< .0001**
nsubjpass	3_22	0.03	0.03	-0.02	0.06	49.71	-0.4	0.72
**nsubjpass**	**9_2**	**0.24**	**0.17**	**0.7**	**0.21**	**49.16**	**3.3**	**0.001**
**nsubj**	**3_22**	**0.11**	**0.06**	**0.64**	**0.06**	**47.57**	**10.62**	**< .0001**
**nsubj**	**4_30**	**0.42**	**0.23**	**0.97**	**0.16**	**97**	**5.93**	**< .0001**
nsubj	17_15	0.22	0.33	-0.66	0.16	97	-4.04	< .001
**nsubj**	**8_1**	**0.25**	**0.09**	**1.61**	**0.2**	**97**	**7.87**	**< .0001**
**nsubj**	**5_18**	**0.17**	**0.12**	**0.65**	**0.19**	**97**	**3.41**	**< .001**
**pobj**	**10_17**	**0.31**	**0.23**	**0.53**	**0.12**	**48.24**	**4.49**	**< .0001**
**pobj**	**3_11**	**0.13**	**0.07**	**0.73**	**0.08**	**47.61**	**9.41**	**< .0001**
**pobj**	**6_3**	**0.2**	**0.08**	**1.16**	**0.12**	**47.96**	**9.49**	**< .0001**
**pobj**	**31_13**	**0.11**	**0.04**	**1.3**	**0.12**	**48.28**	**11.27**	**< .0001**
**pobj**	**12_29**	**0.18**	**0.04**	**1.81**	**0.16**	**48.46**	**11.32**	**< .0001**

Table F7: BERT, lure analysis—Full Model

Relation	Head	Average Attention (Plausible)	Average Attention (Implausible)	*b*	*SE*	*df*	*t*	*p*
dobj	7_9	0.15	0.15	-0.08	0.11	50.85	-0.67	0.25
**dobj**	**6_9**	**0.54**	**0.57**	**-0.23**	**0.12**	**52.46**	**-1.94**	**0.04**
dobj	3_11	0.48	0.39	0.22	0.1	54.13	2.19	0.04
**dobj**	**4_5**	**0.03**	**0.26**	**-2.59**	**0.16**	**53.02**	**-16.68**	**< .0001**
**dobj**	**7_10**	**0.15**	**0.35**	**-1.13**	**0.15**	**53.68**	**-7.44**	**< .0001**
**advmod**	**7_3**	**0.03**	**0.04**	**-0.65**	**0.19**	**53.64**	**-3.5**	**< .001**
**nsubj**	**7_1**	**0.04**	**0.14**	**-1.56**	**0.17**	**95**	**-9.08**	**< .0001**
**nsubj**	**7_11**	**0.03**	**0.13**	**-1.71**	**0.16**	**49.59**	**-10.71**	**< .0001**
**nsubjpass**	**4_10**	**0.07**	**0.13**	**-0.51**	**0.18**	**68.01**	**-2.88**	**0.005**
**nsubjpass**	**8_10**	**0.02**	**0.04**	**-0.83**	**0.29**	**69.77**	**-2.82**	**0.005**
**nsubjpass**	**6_5**	**0.01**	**0.06**	**-1.79**	**0.31**	**69.35**	**-5.87**	**< .0001**
**pobj**	**7_10**	**0.21**	**0.39**	**-1.26**	**0.23**	**49**	**-5.6**	**< .0001**
**pobj**	**4_5**	**0.003**	**0.003**	**-0.15**	**0.06**	**49**	**-2.46**	**0.01**
**pobj**	**8_5**	**0.1**	**0.2**	**-0.87**	**0.15**	**49**	**-5.68**	**< .0001**
**pobj**	**5_0**	**0.002**	**0.002**	**-0.13**	**0.05**	**49**	**-2.69**	**0.007**
**pobj**	**6_9**	**0.003**	**0.005**	**-0.49**	**0.06**	**49**	**-8.28**	**< .0001**
iobj	6_9	0.07	0.09	-0.25	0.15	50.31	-1.61	0.06
iobj	5_10	0.04	0.06	-0.25	0.16	51.26	-1.62	0.06
iobj	3_11	0.15	0.15	0.25	0.13	53.89	1.98	0.06
**iobj**	**3_9**	**0.04**	**0.09**	**-1.02**	**0.21**	**95**	**-4.92**	**< .0001**
**iobj**	**5_9**	**0.003**	**0.02**	**-1.75**	**0.24**	**95**	**-7.25**	**< .0001**

Table F8: BERT, lure analysis—Simple Model

**Relation**	**Head**	**Average Attention (Plausible)**	**Average Attention (Implausible)**	***b***	***SE***	***df***	***t***	***p***
dobj	7_9	0.15	0.15	-0.08	0.1	48.31	-0.74	0.24
dobj	6_9	0.54	0.57	-0.14	0.12	49.38	-1.19	0.13
dobj	3_11	0.48	0.39	0.38	0.11	97	3.43	0.001
**dobj**	**4_5**	**0.03**	**0.26**	**-2.66**	**0.15**	**48.89**	**-17.99**	**< .0001**
**dobj**	**7_10**	**0.15**	**0.35**	**-1.31**	**0.15**	**50.05**	**-8.81**	**< .0001**
**advmod**	**7_3**	**0.03**	**0.04**	**-0.83**	**0.17**	**48.77**	**-4.84**	**< .0001**
**nsubj**	**7_1**	**0.04**	**0.14**	**-1.54**	**0.18**	**97**	**-8.74**	**< .0001**
**nsubj**	**7_11**	**0.03**	**0.13**	**-1.69**	**0.16**	**49.17**	**-10.39**	**< .0001**
**nsubjpass**	**4_10**	**0.07**	**0.13**	**-0.66**	**0.11**	**48.56**	**-6.02**	**< .0001**
**nsubjpass**	**8_10**	**0.02**	**0.04**	**-0.71**	**0.19**	**48.25**	**-3.78**	**< .001**
**nsubjpass**	**6_5**	**0.01**	**0.06**	**-1.63**	**0.19**	**48.2**	**-8.57**	**< .0001**
**pobj**	**7_10**	**0.21**	**0.39**	**-1.26**	**0.23**	**49**	**-5.6**	**< .0001**
**pobj**	**4_5**	**0.003**	**0.003**	**-0.15**	**0.06**	**49**	**-2.46**	**0.01**
**pobj**	**8_5**	**0.1**	**0.2**	**-0.87**	**0.15**	**49**	**-5.68**	**< .0001**
**pobj**	**5_0**	**0.002**	**0.002**	**-0.13**	**0.05**	**49**	**-2.69**	**0.007**
**pobj**	**6_9**	**0.003**	**0.005**	**-0.49**	**0.06**	**49**	**-8.28**	**< .0001**
iobj	6_9	0.07	0.09	-0.18	0.15	49.22	-1.23	0.13
iobj	5_10	0.04	0.06	-0.2	0.15	50.02	-1.33	0.12
iobj	3_11	0.15	0.15	0.1	0.13	50.32	0.75	0.46
**iobj**	**3_9**	**0.04**	**0.09**	**-0.91**	**0.2**	**97**	**-4.59**	**< .0001**
**iobj**	**5_9**	**0.003**	**0.02**	**-1.76**	**0.23**	**97**	**-7.66**	**< .0001**

Table F9: GPT2, lure analysis—Full Model

**Relation**	**Head**	**Average Attention (Plausible)**	**Average Attention (Implausible)**	***b***	***SE***	***df***	***t***	***p***
**dobj**	**2_8**	**0.49**	**0.58**	**-0.35**	**0.11**	**53.09**	**-3.08**	**0.004**
dobj	4_0	0.67	0.68	-0.1	0.13	50.52	-0.72	0.24
dobj	3_8	0.53	0.4	0.36	0.15	95	2.39	0.03
advmod	2_8	0.21	0.17	0.29	0.12	56.39	2.36	0.03
**nsubj**	**4_3**	**0.02**	**0.1**	**-2.55**	**0.22**	**48.22**	**-11.47**	**< .0001**
**nsubj**	**2_9**	**0.53**	**0.57**	**-0.16**	**0.08**	**95**	**-2.03**	**0.03**
**nsubjpass**	**4_3**	**0.03**	**0.08**	**-0.96**	**0.3**	**66.84**	**-3.16**	**0.004**

Table F10: GPT2 – Lure Simple Model

**Relation**	**Head**	**Average Attention (Plausible)**	**Average Attention (Implausible)**	***b***	***SE***	***df***	***t***	***p***
**dobj**	**2_8**	**0.49**	**0.58**	**-0.45**	**0.11**	**49.71**	**-4.01**	**< .001**
dobj	4_0	0.67	0.68	-0.13	0.12	49.33	-1.06	0.15
dobj	3_8	0.53	0.4	0.6	0.16	97	3.74	< .001
advmod	2_8	0.21	0.17	0.2	0.11	48.71	1.91	0.07
**nsubj**	**4_3**	**0.02**	**0.1**	**-2.55**	**0.22**	**48.62**	**-11.6**	**< .0001**
**nsubj**	**2_9**	**0.53**	**0.57**	**-0.16**	**0.08**	**97**	**-2.06**	**0.03**
**nsubjpass**	**4_3**	**0.03**	**0.08**	**-1.22**	**0.19**	**48.23**	**-6.37**	**< .0001**

Table F11: Llama2, lure analysis—Full Model

Relation	Head	Average Attention (Plausible)	Average Attention (Implausible)	*b*	*SE*	*df*	*t*	*p*
**dobj**	**4_11**	**0.18**	**0.24**	**-0.38**	**0.15**	**62.23**	**-2.54**	**0.008**
dobj	3_11	0.18	0.18	-0.004	0.1	62.34	-0.05	0.48
dobj	10_17	0.5	0.3	0.86	0.11	60.22	7.61	< .0001
**dobj**	**22_21**	**0.23**	**0.44**	**-1.32**	**0.22**	**95**	**-5.9**	**< .0001**
**dobj**	**24_10**	**0.21**	**0.35**	**-0.83**	**0.16**	**55.44**	**-5.13**	**< .0001**
**nsubjpass**	**4_30**	**0.03**	**0.19**	**-2.13**	**0.26**	**62.12**	**-8.26**	**< .0001**
nsubjpass	5_18	0.05	0.06	0.19	0.14	50.85	1.38	0.19
**nsubjpass**	**4_28**	**0.02**	**0.06**	**-0.53**	**0.17**	**60.38**	**-3.17**	**0.002**
nsubjpass	3_22	0.04	0.04	-0.12	0.09	55.88	-1.37	0.10
**nsubjpass**	**9_2**	**0.03**	**0.12**	**-0.97**	**0.32**	**60.59**	**-3.07**	**0.002**
**nsubj**	**3_22**	**0.04**	**0.1**	**-0.96**	**0.06**	**48.44**	**-16.63**	**< .0001**
**nsubj**	**4_30**	**0.05**	**0.23**	**-2.21**	**0.2**	**95**	**-11.09**	**< .0001**
**nsubj**	**17_15**	**0.15**	**0.26**	**-0.9**	**0.18**	**95**	**-5.09**	**< .0001**
**nsubj**	**8_1**	**0.19**	**0.41**	**-1.28**	**0.23**	**50.59**	**-5.58**	**< .0001**
**nsubj**	**5_18**	**0.13**	**0.46**	**-1.93**	**0.23**	**48.88**	**-8.48**	**< .0001**

Table F12: Llama2, lure analysis—Simple Model

Relation	Head	Average Attention (Plausible)	Average Attention (Implausible)	*b*	*SE*	*df*	*t*	*p*
**dobj**	**4_11**	**0.18**	**0.24**	**-0.38**	**0.13**	**48.78**	**-2.99**	**0.003**
dobj	3_11	0.18	0.18	0.004	0.09	48.8	0.04	0.96
dobj	10_17	0.5	0.3	0.91	0.1	48.76	9.24	< .0001
**dobj**	**22_21**	**0.23**	**0.44**	**-1.13**	**0.2**	**97**	**-5.57**	**< .0001**
**dobj**	**24_10**	**0.21**	**0.35**	**-0.85**	**0.13**	**48.41**	**-6.61**	**< .0001**
**nsubjpass**	**4_30**	**0.03**	**0.19**	**-2.24**	**0.22**	**97**	**-10.17**	**< .0001**
nsubjpass	5_18	0.05	0.06	-0.07	0.12	49.42	-0.59	0.30
**nsubjpass**	**4_28**	**0.02**	**0.06**	**-0.94**	**0.15**	**50.63**	**-6.3**	**< .0001**
**nsubjpass**	**3_22**	**0.04**	**0.04**	**-0.17**	**0.07**	**49.83**	**-2.34**	**0.01**
**nsubjpass**	**9_2**	**0.03**	**0.12**	**-1.34**	**0.27**	**50.27**	**-4.93**	**< .0001**
**nsubj**	**3_22**	**0.04**	**0.1**	**-0.96**	**0.06**	**48.59**	**-17.39**	**< .0001**
**nsubj**	**4_30**	**0.05**	**0.23**	**-2.21**	**0.2**	**97**	**-11.27**	**< .0001**
**nsubj**	**17_15**	**0.15**	**0.26**	**-0.89**	**0.17**	**97**	**-5.11**	**< .0001**
**nsubj**	**8_1**	**0.19**	**0.41**	**-1.28**	**0.22**	**48.92**	**-5.74**	**< .0001**
**nsubj**	**5_18**	**0.13**	**0.46**	**-1.86**	**0.22**	**48.4**	**-8.53**	**< .0001**

Appendix G

Identifying Syntax-Specialized Attention Heads using Plausible and Implausible Sentences

Stimuli and Procedure for head identification

For each dependency, we created a set of 120 sentences, in minimal pairs (60 plausible and 60 implausible; Table G1). Half the pairs had a particular distance between the words in the critical dependency, and the other half used a different distance, to ensure that identified heads did not simply target a fixed positional offset. We computed accuracy scores for every head in each LLM, using the same approach outlined in Section 2.2 (Table G2). For BERT, we computed accuracy for the dependency in both directions (dependent → head and head → dependent) and tested the single most accurate head.

We report attention heads whose accuracy score for the dependency of interest was at least 10% higher than the fixed offset baseline accuracy of 50% (given that, for each dependency, stimuli included only two possible distances between words in the critical dependency). In some cases, the head with the highest accuracy score assigned less than 1% of its attention weight to the critical dependency token. This was possible because, when computing accuracy scores, we followed Clark et al. (2019) and excluded attention directed to special tokens; such heads assigned the most attention weight to the special token. In these cases (specifically, for Nominal Subject and Passive Nominal Subject in Llama2, and Direct Object in BERT), we instead selected the head with the highest accuracy score whose average attention strength directed from one critical token to the other was at least 0.1 (10% of that token's directed attention). Overall, 9 of the 16 attention heads we identified were the same as those reported in the main analysis.

Table G1

Example localizer stimuli for each dependency (for BERT)

Dependency	Plausible; Distance 1	Plausible; Distance 2	Implausible; Distance 1	Implausible; Distance 2
Direct Object	The teacher **<u>showed</u>** the student a **technique**.	The baker **<u>baked</u>** a **cake**.	The teacher **<u>showed</u>** a technique the **student**.	The baker **<u>baked</u>** the **family**.
Adverb Modifier	The photos from the wedding were **<u>edited</u>** **professionally**.	The artist in the gallery was **<u>viewed</u>** very **admirably**.	The photos from the wedding were **<u>insulted</u>** **professionally**.	The artist in the gallery was **<u>scorned</u>** very **admirably**.
Nominal Subject	The **cat** under the table **<u>chased</u>** the mouse.	The **tailor** **<u>sewed</u>** the dress.	The **table** under the cat **<u>chased</u>** the mouse.	The **telephone** **<u>sewed</u>** the dress.
Passive Nominal Subject	The **story** about the hero was **<u>written</u>** by the author.	The **trophy** was **<u>awarded</u>** by the committee.	The **story** about the hero was **<u>killed</u>** by the author.	The **trophy** was **<u>eaten</u>** by the committee.
Object of Preposition	The truck drove **<u>through</u>** the heavily flooded **intersection**.	The deer bounded **<u>over</u>** the **wall**.	The truck drove **<u>through</u>** the heavily flooded **opinion**.	The deer bounded **<u>over</u>** the **email**.
Indirect Object	The teacher **<u>showed</u>** the **student** a technique.	The baker **<u>baked</u>** the new **customer** a cake.	The teacher **<u>showed</u>** a **technique** the student.	The baker **<u>baked</u>** the new **cake** a customer.

Note: Words in bold constitute the critical dependency. The head of the dependency is underlined (for some dependencies in some LLMs, attention is directed from the head to the dependent; for other cases, attention is directed from the dependent to the head. See Table G2).

Table G2: Attention heads specialized for different dependencies; dependency names are marked with a star if the same attention head was identified as in Section 2.2 of the main manuscript.

BERT

**Relation**	**Head**	**Direction**	**Specialization score**
Direct Object	5-8	head→dep	76.67
Adverb Modifier	5-9	head→dep	99.17
Nominal Subject	5-11	dep→ head	89.17
Passive Nominal Subject	7-10	head→dep	84.17
* Object of Preposition	4-5	head→dep	94.17
* Indirect Object	6-9	dep→head	84.17

GPT-2 small

Relation	Head	Direction	Specialization score
* Direct Object	2-8	dep→head	70.83
Adverb Modifier	3-7	dep→head	100.0
* Nominal Subject	4-3	head→dep	95.0
* Passive Nominal Subject	4-3	head→dep	96.67
* Object of Preposition	4-0	dep→head	81.67

Llama 2

Relation	Hea d	Direction	Specialization score
* Direct Object	4-11	dep→head	98.33
Adverb Modifier	4-11	dep→head	100.0
Nominal Subject	2-27	head→dep	85.0
* Passive Nominal Subject	4-28	head→dep	90.83
* Object of Preposition	3-11	dep→head	99.17

Testing Attention Heads for Penetrability to Plausibility Information

We tested the attention heads identified in this experiment using the same stimuli from the main experiment (Section 2.3.1). Sentences used for head identification versus penetrability testing were different, but had similar or identical syntactic structures. The results are presented in the tables below; significant plausibility effects are in bold.

Table G3: BERT, main analysis—Full Model

Relation	Head	Average Attention (Plausible)	Average Attention (Implausible)	*b*	*SE*	*df*	*t*	*p*
dobj	5-8	0.26	0.24	0.11	0.11	54.06	0.96	0.17
advmod	5-9	0.80	0.79	0.08	0.18	52.73	0.45	0.33
**nsubj**	**5-11**	**0.46**	**0.25**	**1.06**	**0.16**	**48.56**	**6.55**	**<.0001**
**nsubjpass**	**7-10**	**0.35**	**0.21**	**0.74**	**0.30**	**67.48**	**2.5**	**.0076**
**pobj**	**4_5**	**0.01**	**0.003**	**1.09**	**0.24**	**58.61**	**4.54**	**< .0001**
iobj	6_9	0.54	0.57	-0.26	0.12	52.72	-2.18	0.05

Table G4: BERT, main analysis —Simple Model

Relation	Head	Average Attention (Plausible)	Average Attention (Implausible)	*b*	*SE*	*df*	*t*	*p*
dobj	5-8	0.26	0.24	0.065	0.10	50.25	0.63	0.28
advmod	5-9	0.80	0.79	0.24	0.17	47.47	1.39	0.09
nsubj	5-11	**0.46**	**0.25**	**1.08**	**0.17**	**48.54**	**6.28**	**<.0001**
nsubjpass	7-10	**0.35**	**0.21**	**0.94**	**0.17**	**48.18**	**5.50**	**<.0001**
**pobj**	**4_5**	**0.01**	**0.003**	**1.41**	**0.18**	**48.79**	**7.91**	**< .0001**
iobj	6_9	0.54	0.57	-0.16	0.11	49.44	-1.37	0.22

Table G5: GPT-2 Small, main analysis —Full Model

Relation	Head	Average Attention (Plausible)	Average Attention (Implausible)	*b*	*SE*	*df*	*t*	*p*
**dobj**	**2_8**	**0.43**	**0.27**	**0.7**	**0.13**	**53.62**	**5.42**	**< .0001**
advmod	3_7	0.73	0.71	0.07	0.10	51.40	0.662	0.26
**nsubj**	**4_3**	**0.69**	**0.57**	**0.57**	**0.15**	**48.76**	**3.81**	**< .001**
**nsubjpass**	**4_3**	**0.74**	**0.63**	**0.59**	**0.21**	**62.47**	**2.86**	**0.007**
**pobj**	**4_0**	**0.48**	**0.33**	**0.64**	**0.2**	**60.61**	**3.22**	**0.003**

Table G6: GPT-2 Small, main analysis —Simple Model

Relation	Head	Average Attention (Plausible)	Average Attention (Implausible)	*b*	*SE*	*df*	*t*	*p*
**dobj**	**2_8**	**0.43**	**0.27**	**0.8**	**0.13**	**49.86**	**6.33**	**< .0001**
advmod	3_7	0.73	0.71	0.07	0.09	48.75	0.79	0.24
**nsubj**	**4_3**	**0.69**	**0.57**	**0.55**	**0.15**	**48.56**	**3.74**	**< .001**
**nsubjpass**	**4_3**	**0.74**	**0.63**	**0.54**	**0.12**	**48.18**	**4.62**	**< .0001**
**pobj**	**4_0**	**0.48**	**0.33**	**0.72**	**0.16**	**48.24**	**4.54**	**< .0001**

Table G7: Llama2, main analysis —Full Model

Relation	Head	Average Attention (Plausible)	Average Attention (Implausible)	*b*	*SE*	*df*	*t*	*p*
**dobj**	**4_11**	**0.29**	**0.1**	**1.44**	**0.19**	**95**	**7.47**	**< .0001**
advmod	4-11	0.43	0.41	-0.007	0.11	56.79	-0.061	0.48
**nsubj**	**2-27**	**0.12**	**0.11**	**0.13**	**0.05**	**49.46**	**2.75**	**0.004**
**nsubjpass**	**4-28**	**0.18**	**0.09**	**0.49**	**0.19**	**69.26**	**2.58**	**0.006**
**pobj**	**3_11**	**0.13**	**0.07**	**0.6**	**0.09**	**60.45**	**6.68**	**< .0001**

Table G8: Llama2, main analysis —Simple Model

Relation	Head	Average Attention (Plausible)	Average Attention (Implausible)	*b*	*SE*	*df*	*t*	*p*
**dobj**	**4_11**	**0.29**	**0.1**	**1.41**	**0.17**	**97**	**8.24**	**< .0001**
advmod	4-11	0.43	0.41	0.10	0.10	50.09	0.97	0.17
**nsubj**	**2-27**	**0.12**	**0.11**	**0.15**	**0.045**	**48.00**	**3.24**	**0.001**
**nsubjpass**	4-28	**0.18**	**0.09**	**0.90**	**0.14**	**50.64**	**6.51**	**<.0001**
**pobj**	**3_11**	**0.13**	**0.07**	**0.73**	**0.08**	**47.61**	**9.41**	**< .0001**

Table G9: BERT, lure analysis—Full Model

Relation	Head	Average Attention (Plausible)	Average Attention (Implausible)	*b*	*SE*	*df*	*t*	*p*
Direct Object	5-8	0.12	0.15	-0.23	0.10	95.00	-2.25	.013
Adverb Modifier	5-9	0.0025	0.0022	-0.06	0.16	53.01	-0.40	0.35
**Nominal Subject**	**5-11**	**0.07**	**0.19**	**-1.22**	**0.18**	**49.28**	**-6.75**	**<.0001**
**Passive Nominal Subject**	**7-10**	**0.037**	**0.096**	**-1.29**	**0.32**	**66.90**	**-3.99**	**<.0001**
pobj	4_5	0.003	0.003	-0.15	0.06	49	-2.46	0.01
iobj	6_9	0.07	0.09	-0.25	0.15	50.31	-1.61	0.06

Table G10: BERT, lure analysis—Simple Model

Relation	Head	Average Attention (Plausible)	Average Attention (Implausible)	*b*	*SE*	*df*	*t*	*p*
Direct Object	5-8	0.12	0.15	-0.17	0.096	97.00	-1.81	0.037
Adverb Modifier	5-9	0.0025	0.0022	-0.06	0.16	48.66	-0.40	0.35
**Nominal Subject**	**5-11**	**0.07**	**0.19**	**-1.24**	**0.19**	**48.73**	**-6.49**	**<.0001**
**Passive Nominal Subject**	**7-10**	**0.037**	**0.096**	**-1.21**	**0.20**	**48.54**	**-6.07**	**<.0001**
pobj	4_5	0.003	0.003	-0.15	0.06	49	-2.46	0.01
iobj	6_9	0.07	0.09	-0.18	0.15	49.22	-1.23	0.13

Table G11: GPT2, lure analysis—Full Model

Relation	Head	Average Attention (Plausible)	Average Attention (Implausible)	*b*	*SE*	*df*	*t*	*p*
**dobj**	**2_8**	**0.49**	**0.58**	**-0.35**	**0.11**	**53.09**	**-3.08**	**0.004**
advmod	3_7	0.32	0.27	0.22	0.11	54.04	2.00	0.025
**nsubj**	**4_3**	**0.02**	**0.1**	**-2.55**	**0.22**	**48.22**	**-11.47**	**< .0001**
**nsubjpass**	**4_3**	**0.03**	**0.08**	**-0.96**	**0.3**	**66.84**	**-3.16**	**0.004**

Table G12: GPT2 – Lure Simple Model

Relation	Head	Average Attention (Plausible)	Average Attention (Implausible)	*b*	*SE*	*df*	*t*	*p*
**dobj**	**2_8**	**0.49**	**0.58**	**-0.45**	**0.11**	**49.71**	**-4.01**	**< .001**
advmod	**3_7**	0.32	0.27	**0.25**	**0.095**	**48.32**	**2.60**	**0.006**
**nsubj**	**4_3**	**0.02**	**0.1**	**-2.55**	**0.22**	**48.62**	**-11.6**	**< .0001**
**nsubjpass**	**4_3**	**0.03**	**0.08**	**-1.22**	**0.19**	**48.23**	**-6.37**	**< .0001**

Table G13: Llama2, lure analysis—Full Model

Relation	Head	Average Attention (Plausible)	Average Attention (Implausible)	*b*	*SE*	*df*	*t*	*p*
**dobj**	**4_11**	**0.18**	**0.24**	**-0.38**	**0.15**	**62.23**	**-2.54**	**0.008**
advmod	4-11	0.045	0.044	0.24	0.094	46.45	2.58	0.014
nsubj	2-27	**0.049**	**0.049**	0.0026	0.048	49.17	0.054	0.48
**nsubjpass**	**4-28**	**0.02**	**0.06**	**-0.53**	**0.17**	**58.28**	**-3.21**	**0.001**

Table G14: Llama2, lure analysis—Simple Model

Relation	Head	Average Attention (Plausible)	Average Attention (Implausible)	*b*	*SE*	*df*	*t*	*p*
**dobj**	**4_11**	**0.18**	**0.24**	**-0.38**	**0.13**	**48.78**	**-2.99**	**0.003**
advmod	4-11	0.045	0.044	0.098	0.083	49.33	1.18	0.12
nsubj	2-27	0.049	0.049	0.0006	0.044	48.49	0.014	0.49
**nsubjpass**	**4-28**	**0.02**	**0.06**	**-0.94**	**0.15**	**50.63**	**-6.30**	**<.0001**
